# Re-evaluating the potential impact and cost-effectiveness of rotavirus vaccination in 73 Gavi countries: a modelling study

**DOI:** 10.1016/S2214-109X(19)30439-5

**Published:** 2019-11-07

**Authors:** Frédéric Debellut, Andrew Clark, Clint Pecenka, Jacqueline Tate, Ranju Baral, Colin Sanderson, Umesh Parashar, Laura Kallen, Deborah Atherly

**Affiliations:** a PATH, Geneva, Switzerland; b London School of Hygiene & Tropical Medicine, London, UK; c PATH, Seattle, WA, USA; d Centers for Disease Control and Prevention, Atlanta, GA, USA

## Abstract

**Background:**

Previous studies have found rotavirus vaccination to be highly cost-effective in low-income countries. However, updated evidence is now available for several inputs (ie, rotavirus disease mortality rates, rotavirus age distributions, vaccine timeliness, and vaccine efficacy by duration of follow-up), new rotavirus vaccines have entered the market, vaccine prices have decreased, and cost-effectiveness thresholds have been re-examined. We aimed to provide updated cost-effectiveness estimates to inform national decisions about the new introduction and current use of rotavirus vaccines in Gavi countries.

**Methods:**

We calculated the potential costs and effects of rotavirus vaccination for ten successive birth cohorts in 73 countries previously and currently eligible for Gavi support, compared with no vaccination. We used a deterministic cohort model to calculate numbers of rotavirus gastroenteritis cases, outpatient visits, hospitalisations, and deaths between birth and 5 years, with and without rotavirus vaccination. We calculated treatment costs from the government and societal perspectives. The primary outcome measure was the incremental cost-effectiveness ratio (discounted US$ per disability-adjusted life-year averted). Country-specific model input parameters were based on the scientific literature, published meta-analyses, and international databases. We ran deterministic and probabilistic uncertainty analyses.

**Findings:**

Over the period 2018–27, rotavirus vaccination has the potential to prevent nearly 600 000 deaths in Gavi countries. Averted outpatient visits and hospitalisations could lead to treatment savings of approximately $484·1 million from the government perspective and $878·0 million from the societal perspective. The discounted dollars per disability-adjusted life-year averted has a very high probability (>90%) of being less than 0·5 times the gross domestic product per capita in 54 countries, and less than 1·0 times gross domestic product per capita in 63 countries.

**Interpretation:**

Rotavirus vaccination continues to represent good value for money across most Gavi countries despite lower rotavirus mortality estimates and more stringent willingness-to-pay thresholds.

**Funding:**

Bill & Melinda Gates Foundation.

## Introduction

Diarrhoeal diseases are estimated to cause over half a million deaths each year in children younger than 5 years.^1^, ^2^, ^3^ This proportion is roughly 10% of all deaths in this age group, with most deaths occurring in the world's poorest countries. A large proportion (24–37%) of these deaths are estimated to be caused by rotavirus.^4^, ^5^

The introduction of rotavirus vaccines has played an important role in contributing to declines in diarrhoeal mortality and morbidity.^6^ In 2009, WHO recommended introduction of rotavirus vaccination in all national immunisation programmes.^7^ Over 90 countries have introduced the vaccine, with low-income countries benefiting from the financial support of Gavi, the Vaccine Alliance.^8^, ^9^ These introductions have had a profound effect on public health, not just from a rotavirus mortality and morbidity perspective, but also by freeing health-care resources for other priorities in resource-constrained settings.^6^

In addition to the declining diarrhoea burden, the incomes of the world's poorest regions are also growing. For example, between 2005 and 2015, real income per capita increased by nearly 25% in sub-Saharan Africa.^10^ Growing incomes have the potential to increase living standards, reduce poverty, and enable governments to raise additional revenue. Conversely, as incomes grow, countries have less access to international financing mechanisms to support health and development objectives. International donors, such as Gavi, the Global Fund, and the World Bank International Development Association all offer financial support to countries, but this support is linked to country income.^11^, ^12^, ^13^ As a result, countries could face the same challenges with fewer resources to meet them. Furthermore, many of the countries that have yet to introduce rotavirus vaccination are no longer eligible for Gavi funding.^9^, ^14^ In all countries that have received financial support from Gavi, the government is expected to eventually incur the full costs of the programme when Gavi's support expires.

In 2012, Atherly and colleagues^15^ published an impact and cost-effectiveness analysis of rotavirus vaccination in countries eligible for Gavi's support. This, and many other studies, found rotavirus vaccination to be highly cost-effective in low-income and middle-income countries around the world.^16^, ^17^, ^18^, ^19^, ^20^ However, the guidance around cost-effectiveness thresholds used to interpret interventions has changed, calling for the use of more stringent thresholds that better reflect the financial constraints of these countries.^21^ In addition, estimates of rotavirus prevaccination mortality have decreased from 453 000 in 2008 to 215 000 in 2013.^4^, ^22^ Updated evidence is available for rotavirus age distributions, vaccine timeliness, vaccine efficacy, and rotavirus disease treatment costs. The price of rotavirus vaccines has also been decreasing as new products have entered the global market. All these factors are important variables in a cost-effectiveness study. The purpose of this Article is to assess the potential impact and cost-effectiveness of rotavirus vaccination across 73 countries (currently and previously eligible for Gavi support) as a result of these important global trends.

**Research in context**

**Evidence before this study**

Rotavirus is a leading cause of childhood deaths caused by diarrhoea worldwide. Rotavirus vaccines have been available for the past 10 years and introduced in many countries, including countries receiving support from Gavi, the Vaccine Alliance. In countries where they are used, rotavirus vaccines have contributed to the decrease of rotavirus gastroenteritis cases and deaths. We searched PubMed, Google Scholar, and Web of Science from Jan, 2008 to April, 2019 using broad search terms associated with “rotavirus”, “vaccine”, “cost-effective”, “Gavi”, and “low- and middle-income country”. We supplemented identified articles with studies known to the authors. The cost-effectiveness of rotavirus vaccination has been shown in Gavi-eligible countries in several analyses and many analyses in specific low-income and middle-income countries. However, global trends have potentially affected the cost-effectiveness profile of rotavirus vaccines: economic growth has led to a decrease in international support as recipient countries grow wealthier; updated evidence is showing lower rotavirus mortality; the guidance around cost-effectiveness thresholds used to interpret interventions has changed, calling for the use of more stringent thresholds; new products have entered the market; and evidence for several model inputs has been updated.

**Added value of this study**

Our study provides an update on the cost-effectiveness of rotavirus vaccination in previous and current Gavi-eligible countries. We covered more vaccines than in previous studies, including two new, potentially more affordable rotavirus vaccines that entered the market in 2018. The study generates numbers of rotavirus cases, clinic visits, hospitalisations, deaths, and treatment costs averted by vaccination, by country and region. We also calculated costs for vaccination programmes for vaccines with different characteristics.

**Implications of all the available evidence**

Our study provides evidence that rotavirus vaccination is still a cost-effective investment in Gavi countries. Additional rotavirus burden could be averted with more countries adopting the vaccines. Policy makers in countries with reducing international support and who are looking for budget efficiencies should consider newly available products as they might offer more affordable options.

## Methods

### Study design

We examined the projected impact and cost-effectiveness of rotavirus vaccination in 73 countries previously or currently eligible for Gavi support, across all WHO regions (table 1). Results were generated and reported per country and then aggregated per WHO regions and for all Gavi countries. We calculated the potential costs and benefits of nationwide infant rotavirus vaccination, compared with no vaccination, for ten consecutive birth cohorts (2018–27) in 73 Gavi countries.

**Table 1:** Countries considered in the analysis by WHO region and Gavi transition phase (2018)

	**African region**	**Region of the Americas**	**Eastern Mediterranean region**	**European region**	**South-East Asia region**	**Western Pacific region**
Initial self-financing	Benin; Burkina Faso; Burundi; Central African Republic; Chad; Comoros; DR Congo; Eritrea; Ethiopia; Guinea; Guinea-Bissau; Liberia; Madagascar; Malawi; Mali; Mozambique; Niger; Rwanda; Senegal; Sierra Leone; South Sudan*; Tanzania; The Gambia; Togo; Uganda; Zimbabwe	Haiti	Afghanistan; Somalia	..	Nepal; North Korea†	..
Preparatory transition	Cameroon; Côte d'Ivoire; Ghana; Kenya; Lesotho; Mauritania; Zambia	..	Djibouti; Pakistan; Sudan; Yemen	Kyrgyzstan†; Tajikistan	Bangladesh; Myanmar	Cambodia
Accelerated transition	Nigeria; São Tomé and Príncipe	Nicaragua†	..	Uzbekistan	India	Laos; Papua New Guinea; Solomon Islands; Vietnam†
Fully self-financing	Angola; Republic of Congo	Bolivia; Cuba; Guyana; Honduras	..	Armenia†; Azerbaijan; Georgia; Moldova†; Ukraine	Bhutan; Indonesia; Sri Lanka; Timor-Leste	Kiribati; Mongolia†

* Country not included in Atherly et al.^15^

† Countries with medium under-5 mortality over the period 2010–15. Different vaccine efficacy assumptions were applied to countries with medium under-5 mortality and high under-5 mortality over the period 2010–15; all other countries are considered to have high mortality.

Rotavirus gastroenteritis cases, outpatient visits, hospitalisations (hospital admission), deaths, and costs were projected over the first 5 years of life. During the period of analysis, the vaccinated individuals could or could not become ill with rotavirus disease. If they got rotavirus disease, it could be non-severe or severe. Non-severe disease was defined as recovery with or without outpatient care (clinic visit). Severe disease was defined as recovery or death with or without outpatient or inpatient care. We did not consider informal care in this analysis.

Costs and benefits were examined from both the government and societal perspectives and are discounted at 3% per year. Monetary units were presented in 2015 US$. Key outputs of the analysis included aversions of deaths, disability-adjusted life-years (DALYs), cases, hospitalisations and outpatient visits, and health costs as a result of rotavirus vaccination. Additional outputs included the total costs of vaccination and our primary outcome measure, the incremental cost-effectiveness ratio (ICER), expressed as discounted US$ per DALY averted. To allow for comparison of ICERs with a uniform willingness-to-pay threshold that we applied to all countries, gross domestic product (GDP) and population values for each country were used to calculate values for individual countries, regions (WHO regions), and all Gavi countries' GDP per capita. We then used model-generated ICERs and compared the ICER with cost-effectiveness thresholds of 0·5 times and 1·0 times GDP per capita in all examined countries.

### Impact and cost-effectiveness model

We used a Microsoft Excel-based static cohort model with a finely disaggregated age structure (weeks of age up to 5 years) to calculate numbers of rotavirus gastroenteritis cases, clinic visits, hospitalisations, and deaths expected to occur between birth and age 5 years, with and without rotavirus vaccination (UNIVAC version 1.3.41).^23^ Methods used to calculate the direct effects of vaccination have been described in detail elsewhere.^24^, ^25^ In brief, for each week of age, the expected number of disease events (ie cases, visits, hospitalisations, deaths) were multiplied by the expected coverage (adjusted for vaccine timeliness) and efficacy (adjusted for duration of follow-up) of each dose of vaccination. Health-care costs were calculated by multiplying the expected numbers of clinic visits and hospitalisations by the average cost per clinic visit and hospitalisation, from a government and societal perspective. Vaccination costs were calculated by multiplying the total number of doses administered by a wastage factor and other assumptions about price and the costs of delivery. More details on input parameters and values for each country are included later in this Article.

### Disease burden

We estimated approximately 10 000 symptomatic rotavirus gastroenteritis cases per 100 000 children aged younger than 5 years per year on the basis of a global systematic review and meta-analysis by Bilcke and colleagues.^26^ We used WHO region estimates of the proportion of all-cause gastroenteritis cases that are severe (defined as children with moderate or severe dehydration), as a proxy for the proportion of rotavirus gastroenteritis cases that were severe (and non-severe).^27^ To calculate numbers of rotavirus deaths in each country (without vaccination), we estimated means (and 95% CIs) using country-specific estimates from three difference sources (Institute for Health Metrics and Evaluation, Maternal Child Epidemiology Estimation, and WHO US Centers for Diseases Control and Prevention) for the year 2015.^1^, ^3^, ^4^ We elected to use the mean because the range reported by the three different sources was from 158 000 to 202 000 deaths a year in children younger than 5 years for the group of 73 countries. Comparison and discussion of methods and results from the three sources have been published elsewhere.^5^ If a country had already introduced the vaccine in 2015, then the mortality for the most recent prevaccination year was used, using WHO–UNICEF joint estimates of national immunisation coverage to determine the most recent prevaccine year.^28^ In absence of vaccination, we assumed that rotavirus mortality would decrease at the same rate as all-cause mortality for children younger than 5 years of age. Rotavirus age distributions were based on a systematic review and statistical analysis of over 90 hospital datasets.^29^ We assumed that 20% of severe rotavirus gastroenteritis cases would require a hospital admission and further reduced this proportion to account for those without access to hospital, using coverage of the first dose of diphtheria–tetanus–pertussis vaccine (DTP1) as a proxy for access to care. This method generated rates of rotavirus gastroenteritis hospitalisations that were consistent with prevaccination rates previously reported (around 350 per 100 000 per year, among children younger than 5 years).^30^, ^31^, ^32^, ^33^, ^34^, ^35^, ^36^ We assumed that 100% of severe rotavirus gastroenteritis cases and 10% of non-severe cases would require a clinic visit, and again used DTP1 coverage to adjust for access to care. DALY weights were taken from the 2013 Global Burden of Disease study,^37^ using values reported for moderate diarrhoea as a proxy of non-severe rotavirus gastroenteritis and for severe diarrhoea as a proxy of severe rotavirus gastroenteritis. We assumed a duration of illness of 4 days for non-severe rotavirus gastroenteritis and 6 days for severe rotavirus gastroenteritis cases and explored longer and shorter durations in probabilistic analysis.^38^ Input values and ranges for DALY weights and duration of illness are available in the appendix.

### Vaccine preference, coverage, and efficacy

Four rotavirus vaccines prequalified by WHO at end of 2018 were considered in the analysis. These four vaccines were Rotarix (manufactured by GlaxoSmithKline, Rixensart, Belgium), RotaTeq (manufactured by Merck and Co, Kenilworth, NJ, USA), Rotavac (manufactured by Bharat Biotech, Hyderabad, India), and Rotasiil (manufactured by Serum Institute, Pune, India).^39^ Rotarix was administered in a two-dose schedule whereas the other vaccines were administered in a three-dose schedule. Our base-case scenario explored all 73 countries with the vaccine they were using in 2018 for countries already using rotavirus vaccines, and a randomly allocated vaccine (Rotavac or Rotasiil) for countries that were not using rotavirus vaccine at the time of analysis.^9^ This process did not imply a preference for any vaccine but ensured that new products were represented in this analysis. We assumed the use of both Rotavac and Rotasiil in India was a 50–50 distribution countrywide. We also ran so-called what-if scenarios in which all 73 countries used the same product.

Coverage of each dose of rotavirus vaccine is based on the WHO–UNICEF estimates of national immunisation coverage.^28^ The average of DTP1 and DTP3 coverage is used as a proxy for DTP2 coverage. Coverage rates are considered constant throughout the analysis. Data for vaccine coverage timeliness were taken from Clark and colleagues.^25^ Assumptions about vaccine efficacy and waning were based on pooled data from published randomised controlled trials of rotavirus vaccines that are described elsewhere.^40^ In brief, in settings with medium under-5 mortality (defined as 13·5–28·1 deaths per 1000 livebirths) pooled efficacy was 82% (95% credibility interval 74–92%) after 2 weeks of follow-up and 77% (67–84) after 12 months, based on 11 observations. In settings with high under-5 mortality (defined as >28·1 deaths per 1000 livebirths) pooled efficacy was 66% (95% credibility interval 48–81) after 2 weeks of follow-up and 44% (27–59) after 12 months, based on 24 observations. We did not apply any age restriction to the vaccine schedule.^41^

### Vaccine price and delivery costs

Given the importance of Gavi's support to countries, we examined costs of vaccine programmes with and without a Gavi subsidy for the vaccine. The costs with a Gavi subsidy was reflected in the government's perspective as this cost is to the country only. The costs without a Gavi subsidy, representing the cost to countries and to Gavi, were reflected in the societal perspective. As such, the government perspective used each country's cofinancing share based on the Gavi transition policy.^11^, ^42^ The societal perspective reflected each vaccine price: $2·29 per dose for Rotarix ($6·50 for countries procuring through the Pan American Health Organization revolving fund), $3·20 for RotaTeq, $0·85 for Rotavac, and $0·95 for Rotasiil.^43^ The countries that did not introduce rotavirus vaccine when they were still eligible for Gavi support are not automatically accessing Gavi negotiated prices. For these countries, prices were estimated from the WHO vaccine, price, and procurement database (table 2).^44^, ^45^

**Table 2:** Vaccine preference, vaccine price per dose, cofinancing, and vaccine introduction year per country

	**Vaccine preference**	**Vaccine price per dose (US$)**	**Average cofinancing per dose over 2018–27 period (US$)**	**Introduction year**
Afghanistan	Rotarix	2·29	0·20	2018
Angola	Rotarix	2·29	2·29	2014
Armenia	Rotarix	2·29	2·29	2012
Bolivia	Rotarix	6·50	6·50	2008
Burkina Faso	RotaTeq	3·20	0·13	2013
Burundi	Rotarix	2·29	0·20	2013
Cameroon	Rotarix	2·29	0·90	2014
Côte d'Ivoire	RotaTeq	3·20	1·56	2017
Djibouti	Rotarix	2·29	0·53	2014
Eritrea	Rotarix	2·29	0·23	2014
Ethiopia	Rotarix	2·29	0·21	2013
Georgia	Rotarix	2·29	2·29	2013
Ghana	Rotarix	2·29	0·67	2012
Guinea-Bissau	Rotarix	2·29	0·20	2015
Guyana	Rotarix	6·50	6·50	2010
Haiti	Rotarix	6·50	0·20	2013
Honduras	Rotarix	6·50	6·50	2009
India*	Rotavac/Rotasiil	0·85/0·95	0·85/0·95	2017
Kenya	Rotarix	2·29	0·87	2014
Kiribati	Rotarix	6·20	6·20	2015
Lesotho	Rotarix	2·29	0·46	2017
Liberia	Rotarix	2·29	0·20	2016
Madagascar	Rotarix	2·29	0·20	2014
Malawi	Rotarix	2·29	0·20	2012
Mali	RotaTeq	3·20	0·13	2014
Mauritania	Rotarix	2·29	0·55	2014
Moldova	Rotarix	2·29	2·29	2012
Mozambique	Rotarix	2·29	0·20	2015
Nicaragua	Rotarix	6·50	5·98	2006
Niger	Rotarix	2·29	0·20	2014
Pakistan	Rotarix	2·29	1·25	2017
Republic of Congo	Rotarix	2·29	2·29	2014
Rwanda	Rotarix	2·29	0·20	2012
São Tomé and Príncipe	RotaTeq	3·20	2·36	2016
Senegal	Rotarix	2·29	0·20	2014
Sierra Leone	Rotarix	2·29	0·20	2014
Sudan	Rotarix	2·29	1·34	2011
Tajikistan	Rotarix	2·29	0·60	2015
Tanzania	Rotarix	2·29	0·20	2012
The Gambia	Rotarix	2·29	0·20	2013
Togo	Rotarix	2·29	0·20	2014
Uganda	Rotarix	2·29	0·20	2018
Uzbekistan	Rotarix	2·29	2·29	2014
Yemen	Rotarix	2·29	0·68	2012
Zambia	Rotarix	2·29	0·74	2013
Zimbabwe	Rotarix	2·29	0·20	2014

These data are for countries already using rotavirus vaccines at the end of 2018; countries not using rotavirus vaccines at the end of 2018 were randomly allocated one of the newly prequalified vaccines.

* Assuming that 50% of immunised children are receiving Rotavac and the other 50% Rotasiil.

The incremental delivery cost is based on work by the Immunization Costing Action Network. This network completed a systematic review of the cost of immunisation programmes and developed a unit cost repository.^46^ The repository was searched for incremental costs per dose without vaccine cost and returned values for several low-income and lower-middle-income country studies and antigens. We elected to use these values adjusted to 2015 US$: $1·25 for low-income countries and $1·86 for lower-middle-income and upper-middle-income countries. This data input captured all programmatic costs linked to delivering the vaccine, including training costs, staff time, and vaccine storage and distribution. As it covers a wide range of parameters, we varied this data input in probabilistic analysis.

In addition to vaccine price and incremental delivery cost, we accounted for a 5% wastage rate for single dose vaccines and supplies: 10% for Rotasiil and 25% for Rotavac to reflect the multidose presentations. We accounted for the procurement of safety bags with a capacity of 100 doses and a unit cost of $0·80. International handling was estimated at 3·5% of the vaccine price and international transportation at 6·0%.^47^ All inputs used to model cost of the vaccination programme are shown in the appendix.

### Health service costs

Treatment costs for inpatient and outpatient episodes of rotavirus across all 73 Gavi countries were not available. We used modelled estimates of direct medical, direct non-medical, and indirect costs for both inpatient and outpatient episodes. The cost estimation methods are described in detail elsewhere (unpublished). In summary, we generated country-specific direct medical costs using service delivery unit cost estimates from the WHO cost-effectiveness and strategic planning tool (WHO CHOICE)^48^ along with commodity costs. For inpatients costs, we used country-specific estimates of bed day costs at a secondary-level hospital, assuming 4 days of hospital stay, use of six oral rehydration solution packets per day for the duration of hospital stay, and two intravenous solutions. For outpatient costs, WHO CHOICE data for a primary hospital and six packets of oral rehydration solution per day for 2 days were assumed. To estimate direct non-medical costs, we first derived the share of direct medical to direct non-medical costs from the literature. We then used the share of direct non-medical cost from the literature and our estimate of direct medical cost to calculate the direct non-medical cost in each country. Indirect costs were calculated by multiplying the average GDP per capita per day with the average number of days lost to providing care for a patient with diarrhoea. We assumed inpatient caretakers lost one productive day and outpatient caretakers lost a quarter of a productive day based on an unpublished analysis of data from the GEMS study.^49^ Only direct medical costs were used in calculating health service costs from the government perspective. We added direct medical costs, direct non-medical costs, and indirect costs in calculating health-service costs from the societal perspective. Country-specific health-care costs values are available in the appendix.

### Alternative scenarios and probabilistic analysis

In addition to our base-case scenario covering countries with the vaccine they were using in 2018, or a randomly allocated vaccine (Rotavac or Rotasiil) for countries that were not using rotavirus vaccine at the time of analysis, we also explored alternative scenarios looking at the use of Rotarix, Rotavac, and Rotasiil in all Gavi countries. We elected to exclude the use of RotaTeq for non-introducing countries because of the manufacturer's announcement in 2018 to withdraw from the Gavi market.^50^ Inputs for these alternative scenarios are available in the appendix.

We ran probabilistic simulations to account for uncertainty in the parameter inputs. We calculated the proportion of those simulations with an ICER below different possible willingness-to-pay thresholds to indicate the probability that the vaccine would be cost-effective at each threshold. For each country, we generated 1000 runs of results on the basis of randomly selected data inputs using a specified distribution, within a range of low and high values for all study parameters. The complete set of lower and higher input ranges as well as distributions for each input are available in the appendix.

### Role of the funding source

The funder was not involved in the study design, data analysis, interpretation, or reporting of results. The corresponding author had full access to all the data in the study and had final responsibility for the decision to submit for publication.

## Results

Over the period 2018–27 in Gavi countries, without discounting future health benefits, rotavirus vaccination has the potential to avert 158·6 million cases of rotavirus gastroenteritis, 80·7 million outpatient visits, 7·9 million hospitalisations, 576 567 deaths, and 14·7 million DALYs (table 3). Of the cases, visits, and hospitalisations averted, 42% would be in the African region, 41% in the South-East Asian region, and 9% in the Eastern Mediterranean region. Of deaths averted, 65% would be in the African region, 23% in the South-East Asian region, and 10% in the Eastern Mediterranean region.

**Table 3:** Health and economic benefits over a 10-year period (2018–27) for the base-case scenario

	**African region**	**Region of the Americas**	**Eastern Mediterranean region**	**European region**	**South-East Asia region**	**Western Pacific region**	**All Gavi countries**
**Averted rotavirus burden**							
Cases	64 941 257	2 279 644	14 727 951	3 514 830	66 895 132	6 202 581	158 561 393
Visits	23 883 590	938 830	5 922 875	859 706	15 531 821	1 674 650	48 811 472
Hospitalisations	2 826 609	78 392	392 566	93 906	2 096 962	244 059	5 732 494
Deaths	376 560	3293	57 927	2547	130 824	5417	576 567
DALYs*	9 407 363	89 129	1 488 332	73 308	3 472 376	152 446	14 682 955
**Averted health-care costs (US$)**							
Government perspective*	192 412 959	15 216 757	26 438 853	14 047 862	186 275 477	49 726 662	484 118 569
Societal perspective*	351 173 250	26 076 818	51 733 623	24 427 446	345 072 153	79 549 803	878 033 093
**Vaccine programme costs (US$)**							
With Gavi subsidy* (cost to country only)	1 211 762 128	105 071 230	375 178 351	93 259 104	2 360 479 712	208 622 785	4 354 373 309
Without Gavi subsidy* (cost to country and to Gavi)	2 187 656 303	129 326 733	550 683 257	104 171 153	2 459 017 468	217 671 893	5 648 526 807
**Cost per DALY averted (US$)**							
Government perspective*†	108 (29–568)	1008 (71–3389)	234 (42–463)	1081 (196–5396)	626 (242–4529)	1042 (137–2661)	264 (202–428)
Societal perspective*	195	1158	335	1088	609	906	325
Cost per DALY averted (government perspective) as a proportion of GDP per capita‡	0·09	0·30	0·17	0·50	0·33	0·49	0·16

DALYs=disability-adjusted life-years. GDP=gross domestic product.

* Discounted values.

† Figures in parentheses show 95% uncertainty intervals (2·5th and 97·5th percentiles of 1000 simulations).

‡ GDP per capita in current US$ calculated for each region.

In terms of economic benefits, outpatient visits and hospitalisations averted represent $484·1 million from the government perspective and $878·0 million from the societal perspective (table 3). Most of the costs from the government perspective are averted in the African region, with 40%, 38% in the South-East Asia region, and 10% in the Western Pacific region. The total vaccination programme cost across all countries is estimated to be $4·4 billion, assuming a Gavi subsidy to countries, and about $5·6 billion without considering Gavi subsidy on vaccine prices. The regional distribution of this cost also differs depending on the inclusion or exclusion of Gavi support, reflecting regions where countries are receiving more support. Without cofinancing, 44% of the global vaccine programme cost would be in the South-East Asia region, 39% in the African region, and 10% in the Eastern Mediterranean region. Accounting for Gavi subsidy, 54% of the cost is attributed to countries in the South-East Asia region, 28% in the African region, and 9% in the Eastern Mediterranean region.

**Figure 1:**
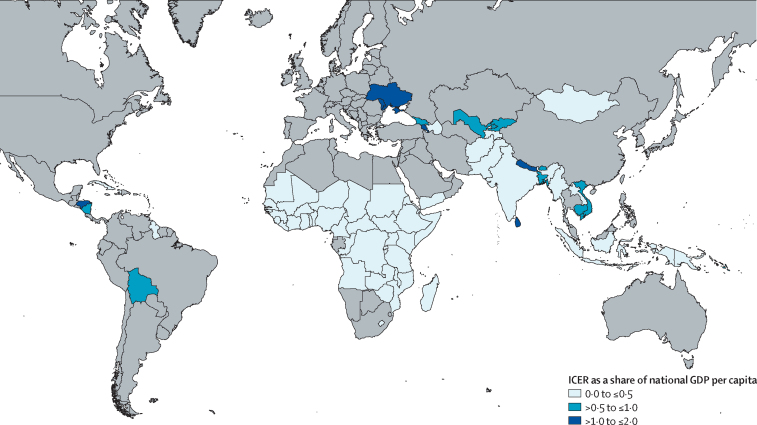
Map displaying country ICER as a share of GDP per capita from the government perspective GDP=gross domestic product. ICER=incremental cost-effectiveness ratio.

From the societal perspective, incremental cost-effectiveness ratios expressed in US$ per DALY averted ranged from $195 for the African region to $1158 for the region of the Americas. Overall from the societal perspective, the cost per DALY averted in Gavi countries is $325. From the government perspective, the cost per DALY averted ranges from $108 for the African region to $1081 for the European region. Overall from the government perspective, the cost per DALY averted in Gavi countries is $264 (figure 1, Table 3, Table 4).

**Table 4:** Results by country in the base case scenario (all countries using rotavirus vaccine from 2018 to 2027)

		**Number of fully immunised children**	**Averted cases of rotavirus**	**Averted deaths by rotavirus**	Total health-care costs averted government perspective (US$)*	Vaccine programme costs (US$)*	Vaccine programme costs with Gavi subsidy (US$)*	Cost per DALY averted government perspective (US$)*†	**GDP per capita (US$)**
Afghanistan	Rotarix	7 762 176	1 580 540	7305	1 790 959	54 638 902	20 878 029	102 (68–174)	562
Angola	Rotarix	9 358 710	2 242 237	30 528	21 333 511	77 669 464	77 669 464	74 (36–119)	3309
Armenia	Rotarix	307 794	106 670	9	473 387	2 499 384	2 499 384	4337 (2225–6420)	3615
Azerbaijan	Rotavac	1 296 082	256 505	183	1 388 389	10 954 903	10 954 903	1824 (678–3460)	3879
Bangladesh	Rotasiil	27 821 356	6 381 993	6038	12 508 907	224 858 236	168 951 095	911 (565–1219)	1359
Benin	Rotavac	3 409 188	915 805	6618	2 419 236	23 113 488	13 917 319	69 (37–100)	789
Bhutan	Rotavac	130 982	31 051	16	130 410	1 101 703	1 101 703	1993 (851–3758)	2774
Bolivia	Rotarix	2 458 502	552 970	820	4 601 265	40 490 928	40 490 928	1599 (868–2513)	3105
Burkina Faso	Rotateq	6 938 849	1 741 437	11 105	4 990 861	92 210 089	26 332 567	76 (30–134)	627
Burundi	Rotarix	4 369 163	975 496	6008	1 609 641	30 075 387	11 492 083	66 (35–97)	286
Cambodia	Rotasiil	3 093 143	693 787	646	1 760 655	25 019 452	17 315 754	855 (480–1222)	1270
Cameroon	Rotarix	7 659 052	1 813 529	13 867	7 230 672	61 673 034	39 524 515	93 (55–128)	1375
Central African Republic	Rotavac	733 714	269 033	3419	421 169	6 240 284	3 628 809	38 (16–68)	382
Chad	Rotasiil	2 812 216	766 128	12 178	1 373 576	21 493 685	12 650 864	39 (20–61)	664
Comoros	Rotasiil	228 838	59 906	194	154 074	1 534 044	902 903	150 (77–227)	775
Côte d'Ivoire	Rotateq	7 677 006	2 171 684	9889	22 224 658	122 376 764	79 727 547	239 (141–336)	1535
Cuba	Rotasiil	1 116 234	508 721	19	6 191 925	8 998 257	8 998 257	1681 (98–4332)	7602
DR Congo	Rotasiil	27 297 657	7 268 535	53 013	18 669 915	177 176 708	104 282 116	64 (28–98)	449
Djibouti	Rotarix	178 289	40 238	121	127 781	1 441 579	786 340	212 (125–304)	1862
Eritrea	Rotarix	1 539 050	261 276	770	421 978	10 611 584	4 155 848	188 (91–303)	583
Ethiopia	Rotarix	26 078 179	5 162 506	16 375	7 580 039	187 304 858	72 057 918	152 (85–217)	707
Georgia	Rotarix	421 489	192 937	12	738 532	3 395 328	3 395 328	3581 (1805–5246)	3866
Ghana	Rotarix	8 085 885	1 775 985	6344	6 160 965	64 090 055	37 213 746	192 (110–265)	1513
Guinea	Rotavac	2 600 878	695 933	2897	1 178 462	18 533 035	10 777 108	130 (77–184)	662
Guinea-Bissau	Rotarix	584 936	127 634	848	320 995	4 118 150	1 573 583	59 (25–96)	642
Guyana	Rotarix	141 621	30 965	46	223 693	2 335 070	2 335 070	1736 (1303–2282)	4529
Haiti	Rotarix	1 642 083	380 229	1557	609 246	27 623 504	4 702 378	103 (50–157)	740
Honduras	Rotarix	1 920 553	422 385	391	2 083 282	31 797 350	31 797 350	2667 (1978–3549)	2361
India	Rotavac/Rotasiil	210 355 220	46 083 379	106 007	117 772 708	1 761 981 734	1 761 981 734	588 (428–747)	1710
Indonesia	Rotasiil	35 606 711	8 613 368	11 969	41 943 398	320 037 163	320 037 163	866 (463–1306)	3570
Kenya	Rotarix	14 630 923	3 225 501	8877	8 233 918	117 831 196	73 903 636	283 (182–383)	1455
Kiribati	Rotarix	25 503	5 365	14	23 395	406 410	406 410	1080 (646–1646)	1587
Kyrgyzstan	Rotavac	1 219 828	466 414	232	2 902 866	10 203 078	7 256 136	625 (147–1107)	1078
Laos	Rotasiil	1 066 947	265 434	1481	2 476 970	9 945 424	9 205 425	177 (99–243)	2339
Lesotho	Rotarix	550 446	126 078	594	403 802	4 419 060	2 325 045	133 (79–178)	1040
Liberia	Rotarix	1 481 187	352 291	1278	587 456	10 701 360	4 089 089	106 (56–164)	455
Madagascar	Rotarix	7 244 209	1 622 511	6370	2 465 080	50 490 355	19 292 831	102 (45–176)	402
Malawi	Rotarix	6 138 614	1 487 654	6147	3 486 309	42 520 140	16 247 338	81 (38–121)	300
Mali	Rotateq	5 370 633	1 528 415	7623	2 536 699	80 689 518	23 042 622	107 (42–197)	780
Mauritania	Rotarix	1 200 458	270 702	1537	870 930	9 887 444	5 457 313	118 (72–166)	1102
Mongolia	Rotavac	598 514	234 615	133	976 855	4 904 458	4 904 458	997 (491–1628)	3694
Mozambique	Rotarix	10 006 557	2 270 541	9103	4 751 912	70 827 392	27 063 800	97 (49–149)	382
Myanmar	Rotavac	8 102 780	2 040 478	4888	4 479 595	68 695 435	49 153 966	351 (351–215)	1196
Nepal	Rotavac	4 864 133	1 116 376	816	1 527 722	33 107 970	19 252 409	751 (370–1190)	729
Nicaragua	Rotarix	1 092 186	384 373	460	1 507 347	18 081 624	16 747 246	1186 (828–1596)	2151
Niger	Rotarix	8 811 793	2 098 720	19 320	3 657 328	63 969 987	24 443 523	43 (21–69)	364
Nigeria	Rotavac	35 492 367	10 650 771	91 934	28 485 738	342 230 664	287 705 916	116 (69–169)	2176
North Korea	Rotavac	3 272 406	1 214 173	779	2 212 329	22 063 877	12 830 293	467 (231–735)	..
Pakistan	Rotarix	37 967 843	8 098 797	23 446	12 209 306	308 853 983	222 737 186	343 (165–567)	1444
Papua New Guinea	Rotavac	1 607 485	441 152	1145	1 412 872	14 741 493	14 223 924	432 (298–599)	2500
Moldova	Rotarix	319 744	114 889	9	827 754	2 576 006	2 576 006	3433 (1681–5255)	1900
Republic of Congo	Rotarix	1 536 206	348 359	1090	4 065 847	12 290 418	12 290 418	292 (97–560)	1528
Rwanda	Rotarix	3 585 413	715 749	2539	2 031 404	24 601 057	9 419 779	112 (37–207)	703
São Tomé and Príncipe	Rotateq	66 017	15 146	27	169 848	969 103	790 346	729 (453–1378)	1715
Senegal	Rotarix	5 356 154	1 078 428	3025	3 505 164	36 891 487	14 096 578	134 (53–217)	953
Sierra Leone	Rotarix	2 237 531	494 152	4068	1 096 614	15 995 382	6 111 983	51 (26–78)	505
Solomon Islands	Rotasiil	168 506	39 695	35	179 991	1 363 103	1 275 262	1116 (604–1768)	2005
Somalia	Rotasiil	2 731 238	791 252	7413	926 056	19 683 313	11 585 139	58 (31–90)	434
South Sudan	Rotasiil	1 160 690	362 015	2364	615 357	8 780 072	5 167 745	77 (34–135)	759
Sri Lanka	Rotavac	2 868 156	1 315 209	69	4 936 522	23 848 677	23 848 677	3938 (1801–6603)	3835
Sudan	Rotarix	12 845 274	2 833 307	14 567	8 823 965	103 124 331	77 184 096	184 (121–245)	2415
Tajikistan	Rotarix	2 291 676	515 367	1320	995 629	18 273 646	10 308 538	265 (159–371)	796
Gambia	Rotarix	818 934	167 343	533	330 064	5 663 362	2 164 023	137 (41–276)	473
Timor-Leste	Rotasiil	391 577	99 103	242	763 884	3 322 673	3 322 673	400 (163–726)	1405
Togo	Rotarix	2 416 422	540 639	3061	1 293 933	16 683 379	6 374 873	67 (27–110)	578
Uganda	Rotarix	15 696 079	3 497 987	9314	7 773 885	110 814 276	42 343 158	146 (76–212)	580
Ukraine	Rotasiil	630 398	692 799	35	3 911 990	10 290 365	10 290 365	2609 (812–4 702)	2072
Tanzania	Rotarix	22 592 798	5 235 956	12 673	12 913 994	154 758 157	66 128 551	160 (75–255)	852
Uzbekistan	Rotarix	5 772 626	1 169 250	747	2 809 315	45 978 443	45 978 443	1979 (532–4606)	2111
Vietnam	Rotavac	14 251 963	4 522 533	1963	42 895 925	161 291 552	161 291 552	1930 (410–5174)	2171
Yemen	Rotarix	6 182 800	1 383 818	5075	2 560 785	62 941 149	42 007 560	303 (160–471)	990
Zambia	Rotarix	6 573 203	1 560 484	6263	4 279 137	66 609 961	45 525 340	259 (196–316)	1270
Zimbabwe	Rotarix	4 669 296	1 044 688	4766	2 768 787	41 811 906	21 871 830	159 (109–210)	1029

DALY=disability-adjusted life-year. GDP=gross domestic product.

* Discounted value.

† Figures in parentheses show 95% uncertainty intervals (2·5th and 97·5th percentiles of 1000 simulations).

From the government perspective, regional ICERs represent only a small share of the GDP per capita, ranging from 0·09 times GDP per capita in the African region to 0·50 times GDP per capita in the European region. Across Gavi countries, the cost per DALY averted is approximately 0·16 times GDP per capita.

**Figure 2:**
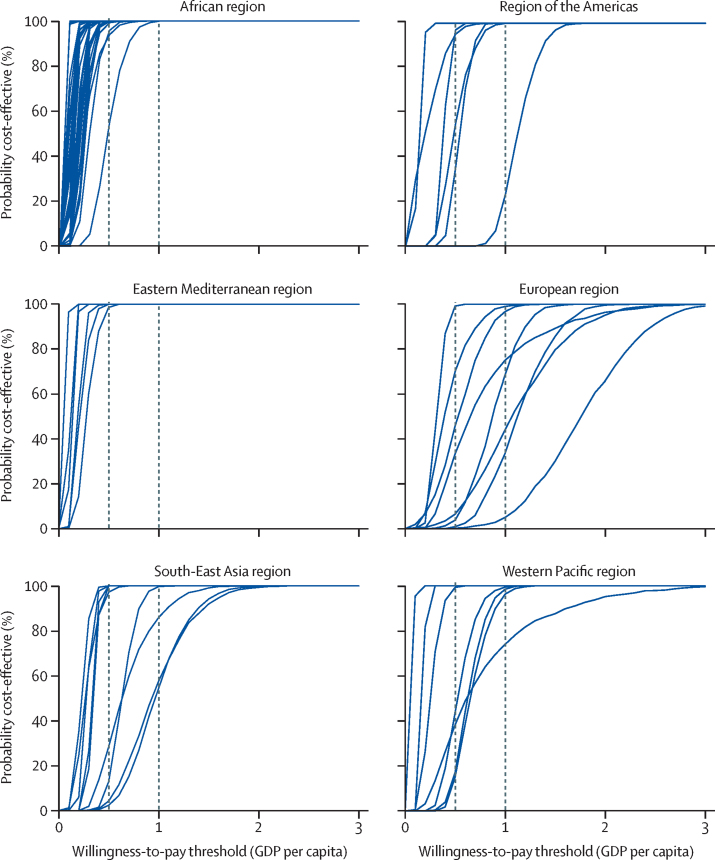
Cost-effectiveness acceptability curves for 72 Gavi countries,* displayed per WHO region Curves are country-specific and show the probability in the base-case scenario for rotavirus vaccination to be cost-effective from the government perspective (accounting for Gavi subsidy) at different thresholds. Vertical dotted lines represent thresholds of 0·5 times and 1·0 times GDP per capita. GDP=gross domestic product. *North Korea was excluded because of the absence of data for GDP per capita.

Results from the probabilistic analysis show that there is a very high probability (>90%) that the discounted US$ per DALY averted will be less than 0·5 times the national GDP per capita in 54 countries and less than 1·0 times GDP per capita in 63 countries. Countries where the probability of rotavirus vaccination being cost-effective is the lowest are in the Americas, Europe, and Western Pacific, which is consistent with the results of our deterministic analysis (figure 2).

Four countries stand out as having less than 50% probability of rotavirus vaccine being cost-effective at 1·0 times GDP per capita threshold: Armenia, Honduras, Moldova, and Ukraine. These four countries are fully self-financing countries. The appendix contains the results of our alternative scenarios, showing ICERs for all different vaccines in each country.

## Discussion

Rotavirus vaccination is an impactful and cost-effective intervention for a disease that causes around 200 000 deaths in children younger than 5 years each year. This analysis serves as an important reminder to continue to prioritise immunisation in the context of efforts to achieve universal health coverage, health equity, and other important priorities. Immunisation should continue to be an essential component of these efforts, especially for countries that face decreasing international financial assistance and pressure to achieve additional objectives.

In previous studies, the cost per DALY averted for all Gavi-supported countries was reported to be $42.^15^, ^16^ Although the ICERs presented here are higher than in previous analyses, rotavirus vaccination is likely to be cost-effective across most Gavi countries, even in countries not receiving any support and not accessing lower, Gavi-like vaccine prices. Comparison with previous studies is not straightforward because of differing methods and assumptions; however, results are in line with the various trends affecting rotavirus cost-effectiveness, including decreases in rotavirus mortality and reduced donor support. A comparison of the results presented in this Article and the previous analysis^15^ shows that the largest changes in ICERs across Gavi countries are due to changes in rotavirus burden estimates and increases in the prices countries pay for vaccines as they transition from Gavi support.

Like previous analyses, this analysis also shows that the health benefits of rotavirus vaccination are concentrated in the highest-burden regions.^15^, ^16^ In addition, many of the countries that are most quickly transitioning from Gavi support are also the ones with a lower burden of disease. Unsurprisingly, rotavirus vaccination is less cost-effective in some lower-burden regions that pay higher vaccine prices (Figure 1, Figure 2). The geographies in which rotavirus vaccination is least cost-effective because of a lower burden and higher vaccine costs are those that appear to benefit most from the availability of new rotavirus vaccine products with lower prices. Such products are appearing on the market as the burden is falling and country vaccine costs are rising, and as countries shoulder a larger share of their vaccine costs, these new lower-cost vaccine products have the potential to reduce or to mitigate the effects of declining international support. A full product comparison by country is beyond the scope of this analysis, but additional analyses might illustrate economic benefits for lower-cost products in countries with less access to Gavi support.

Finally, this analysis and comparison to previous work is being undertaken in the context of evolving guidance on cost-effectiveness thresholds. Previous cost-effectiveness analyses have relied on the guidance from the WHO World Health Report, using 3·0 times the GDP per capita as a threshold to characterise cost-effective interventions, and 1·0 times the GDP per capita for highly cost-effective interventions.^51^ This guidance has been updated since 2012, highlighting the need to account for additional dimensions when framing cost-effectiveness results such as affordability, feasibility, and other country-specific factors.^52^ Attempts to refine these norms have resulted in more stringent thresholds.^53^ Although we were unable to apply a country-contextualised threshold in a global analysis, we did apply more stringent willingness-to-pay thresholds. Vaccination has always been considered one of the best buys in public health and the evolving norms in interpreting cost-effectiveness results have not fundamentally changed the outcome of our analysis. Rotavirus vaccination still represents good, if not excellent, value for money, which is an important message for donors such as Gavi and country governments.

This analysis includes several limitations worth noting. First, we used a transparent and widely used static cohort model to estimate only the direct effect among vaccinated children. Excluding other indirect (herd immunity) effects is likely to underestimate the potential impact and value of rotavirus vaccines, so results from this Article should be viewed as conservative estimates. Second, this is a global analysis. Although we explored rotavirus vaccination for 73 countries, several input values used for modelling were average values at a global or regional level and not country-specific, so results should be interpreted cautiously. A country study involving detailed country engagement would probably yield improved data inputs leading to more accurate results. Third, several data inputs are uncertain. In the absence of a reliable measure of treatment-seeking rates and access to care, we used proportions of cases seeking care and DTP1 coverage rate as an indicator for access to care and ran a plausibility check against the limited country-specific data available in the published literature. Further, although we searched a comprehensive database to inform cost estimates of incremental vaccine programmes, data were not available for many countries and we were not able to differentiate these costs by vaccine product.^46^ In addition, we projected each country's Gavi eligibility status into the future based on current Gavi status, the Gavi transition policy, and projected International Monetary Fund growth rates. Although we believe our projections are reasonable, economic growth is difficult to project and deviations from projections will influence Gavi transitions, vaccine prices, and country-specific results. Finally, this Article addresses the impact and cost-effectiveness of rotavirus vaccination. Although value for money is a crucial consideration, affordability is also essential and can be examined through a budget impact analysis. Although crucial to decision making for a country, budget impacts are beyond the scope of this analysis. However, our finding that rotavirus vaccine is likely to be less cost-effective in countries with less international support highlights the importance of affordability and the need for such analyses.

Overall, rotavirus vaccination offers strong value for money across Gavi countries despite important global trends contributing to higher cost-effectiveness ratios. Countries transitioning away from Gavi support should explore newly prequalified vaccines as an option that might provide enhanced value for money. Countries that have yet to introduce rotavirus vaccination should actively consider the potential benefits and cost-effectiveness of rotavirus vaccination as a step to achieving broader health goals.
