# Supplementary material for: Re-evaluating the potential impact and cost-effectiveness of rotavirus vaccination in 73 Gavi countries: a modelling study
Source: Lancet Glob Health. 2019 Nov 7;7(12):e1664–74. doi: 10.1016/S2214-109X(19)30439-5 (PMC7024955; doi:10.1016/S2214-109X(19)30439-5)
Supplement: Supplementary appendix [file mmc1.pdf]

THE LANCET
Global Health

Supplementary appendix

This appendix formed part of the original submission and has been peer reviewed. We post it as supplied by the authors.

Supplement to: Debellut F, Clark A, Pecenka C, et al. Re-evaluating the potential impact and cost-effectiveness of rotavirus vaccination in 73 Gavi countries: a modelling study. *Lancet Glob Health* 2019; **7:** e1664–74.

Supplementary materials

1. Pre-vaccination disease event rates per 100,000 population per year among children under 5 years of age

	RVGE deaths*			Non-severe RVGE cases			Severe RVGE cases			Non-severe RVGE clinic visits			Severe RVGE clinic visits			Severe RVGE hospital admissions		
	**Mid**	*Low*	*High*	**Mid**	*Low*	*High*	**Mid**	*Low*	*High*	**Mid**	*Low*	*High*	**Mid**	*Low*	*High*	**Mid**	*Low*	*High*
**All 73 countries**																		
Median	39	26	58	7,807	5,583	10,907	2,193	1,417	3,093	718	258	2,523	1,889	1,234	2,641	378	123	792
Min	0	0	1	6,837	5,222	8,109	1,045	457	1,872	412	149	1,386	988	423	1,425	198	42	427
Max	187	126	317	8,955	6,543	12,128	3,163	1,778	5,891	887	322	3,000	2,907	1,742	4,715	581	174	1,415
Afghanistan	59	38	93	8,020	5,676	11,436	1,980	1,324	2,564	605	214	2,156	1,493	998	1,933	299	100	580
Angola	159	97	259	7,414	5,275	10,626	2,586	1,725	3,374	629	224	2,253	2,193	1,463	2,861	439	146	858
Armenia	1	1	2	8,868	6,435	11,933	1,132	565	2,067	866	314	2,912	1,105	552	2,018	221	55	605
Azerbaijan	8	3	24	8,955	6,543	11,823	1,045	457	2,177	847	309	2,796	988	432	2,060	198	43	618
Bangladesh	13	9	17	8,196	5,898	11,431	1,804	1,102	2,569	805	290	2,806	1,772	1,082	2,523	354	108	757
Benin	83	76	91	7,353	5,258	10,499	2,647	1,742	3,501	637	228	2,273	2,292	1,508	3,032	458	151	910
Bhutan	7	2	20	8,498	6,059	11,915	1,502	941	2,085	824	294	2,889	1,457	913	2,023	291	91	607
Bolivia	20	10	41	8,240	5,956	11,358	1,760	1,044	2,642	806	291	2,777	1,721	1,021	2,584	344	102	775
Burkina Faso	81	48	136	6,970	5,411	8,973	3,030	1,589	5,027	654	254	2,104	2,842	1,491	4,715	568	149	1,415
Burundi	74	61	90	7,549	5,325	10,879	2,451	1,675	3,121	743	262	2,676	2,411	1,648	3,071	482	165	921
Cambodia	14	9	21	7,819	5,564	11,198	2,181	1,436	2,802	726	258	2,598	2,024	1,333	2,600	405	133	780
Cameroon	96	74	124	7,131	5,355	9,644	2,869	1,645	4,356	662	248	2,237	2,663	1,527	4,043	533	153	1,213
Central African Republic	155	75	317	7,181	5,310	9,906	2,819	1,690	4,094	444	164	1,531	1,742	1,044	2,530	348	104	759
Chad	187	126	276	7,324	5,235	10,470	2,676	1,765	3,530	417	149	1,492	1,525	1,006	2,012	305	101	604
Comoros	37	31	45	7,494	5,224	10,921	2,506	1,776	3,079	670	234	2,441	2,241	1,587	2,753	448	159	826
Congo	39	18	85	7,222	5,370	9,880	2,778	1,630	4,120	657	244	2,248	2,528	1,484	3,749	506	148	1,125
Côte d'Ivoire	59	48	72	7,536	5,274	10,939	2,464	1,726	3,061	671	235	2,434	2,193	1,536	2,725	439	154	817
Cuba	0	0	1	8,150	5,695	11,665	1,850	1,305	2,335	815	285	2,916	1,850	1,305	2,335	370	131	701
Dem. People's Republic of Korea	9	7	12	8,306	5,833	11,861	1,694	1,167	2,139	787	276	2,811	1,606	1,107	2,027	321	111	608
Democratic Republic of the Congo	90	70	117	6,837	5,521	8,109	3,163	1,479	5,891	543	219	1,610	2,512	1,174	4,677	502	117	1,403
Djibouti	34	25	45	8,071	5,700	11,522	1,929	1,300	2,478	717	253	2,558	1,713	1,154	2,201	343	115	660
Eritrea	45	26	78	7,585	5,313	10,991	2,415	1,687	3,009	737	258	2,671	2,347	1,640	2,925	469	164	877
Ethiopia	44	37	53	7,818	5,759	10,659	2,182	1,241	3,341	639	236	2,180	1,785	1,015	2,733	357	102	820
Gambia	36	14	96	8,220	6,335	9,850	1,780	665	4,150	812	313	2,433	1,759	657	4,100	352	66	1,230
Georgia	1	1	1	8,874	6,442	11,927	1,126	558	2,073	887	322	2,982	1,126	558	2,073	225	56	622
Ghana	43	33	56	7,022	5,411	9,141	2,978	1,589	4,859	667	257	2,171	2,830	1,510	4,616	566	151	1,385
Guinea	60	59	62	7,360	5,261	10,510	2,640	1,739	3,490	551	197	1,965	1,975	1,300	2,610	395	130	783
Guinea-Bissau	81	56	118	7,254	5,321	10,090	2,746	1,679	3,910	688	252	2,391	2,603	1,592	3,707	521	159	1,112
Guyana	17	14	20	7,982	5,692	11,226	2,018	1,308	2,774	785	280	2,762	1,986	1,287	2,730	397	129	819
Haiti	48	37	63	8,549	6,364	11,054	1,451	636	2,946	718	267	2,321	1,219	535	2,475	244	53	742

Honduras	11	*9*	*14*	8,128	*5,737*	*11,530*	1,872	*1,263*	*2,470*	805	*284*	*2,854*	1,853	*1,251*	*2,445*	371	*125*	*734*
India	29	*27*	*32*	8,393	*5,889*	*11,979*	1,607	*1,111*	*2,021*	745	*261*	*2,659*	1,427	*987*	*1,795*	285	*99*	*538*
Indonesia	17	*10*	*30*	7,817	*5,825*	*10,474*	2,183	*1,175*	*3,526*	755	*281*	*2,530*	2,108	*1,135*	*3,406*	422	*113*	*1,022*
Kenya	33	*25*	*44*	7,751	*5,628*	*10,791*	2,249	*1,372*	*3,209*	746	*271*	*2,595*	2,164	*1,320*	*3,087*	433	*132*	*926*
Kiribati	29	*16*	*53*	8,369	*6,019*	*11,704*	1,631	*981*	*2,296*	783	*282*	*2,739*	1,527	*918*	*2,149*	305	*92*	*645*
Kyrgyzstan	6	*4*	*9*	8,722	*6,204*	*12,128*	1,278	*796*	*1,872*	848	*302*	*2,947*	1,242	*774*	*1,820*	248	*77*	*546*
Lao People's Democratic Republic	74	*70*	*78*	7,701	*5,578*	*10,856*	2,299	*1,422*	*3,144*	668	*242*	*2,356*	1,995	*1,234*	*2,729*	399	*123*	*819*
Lesotho	64	*57*	*71*	7,713	*5,583*	*10,786*	2,287	*1,417*	*3,214*	753	*272*	*2,632*	2,233	*1,383*	*3,137*	447	*138*	*941*
Liberia	52	*40*	*67*	7,484	*5,228*	*10,888*	2,516	*1,772*	*3,112*	642	*224*	*2,336*	2,159	*1,520*	*2,670*	432	*152*	*801*
Madagascar	55	*28*	*110*	7,658	*5,456*	*10,907*	2,342	*1,544*	*3,093*	614	*219*	*2,187*	1,879	*1,238*	*2,481*	376	*124*	*744*
Malawi	49	*39*	*61*	7,412	*5,223*	*10,719*	2,588	*1,777*	*3,281*	722	*254*	*2,610*	2,520	*1,731*	*3,196*	504	*173*	*959*
Mali	64	*29*	*139*	8,157	*6,290*	*9,835*	1,843	*710*	*4,165*	684	*264*	*2,060*	1,545	*595*	*3,490*	309	*60*	*1,047*
Mauritania	63	*50*	*79*	7,394	*5,230*	*10,659*	2,606	*1,770*	*3,341*	667	*236*	*2,404*	2,351	*1,597*	*3,013*	470	*160*	*904*
Mongolia	7	*4*	*15*	8,085	*5,841*	*11,321*	1,915	*1,159*	*2,679*	797	*288*	*2,791*	1,889	*1,143*	*2,641*	378	*114*	*792*
Mozambique	47	*36*	*62*	7,473	*5,224*	*10,870*	2,527	*1,776*	*3,130*	685	*239*	*2,489*	2,315	*1,627*	*2,867*	463	*163*	*860*
Myanmar	27	*20*	*36*	7,807	*5,824*	*10,444*	2,193	*1,176*	*3,556*	706	*263*	*2,360*	1,982	*1,063*	*3,215*	396	*106*	*965*
Nepal	10	*6*	*17*	8,604	*6,243*	*11,760*	1,396	*757*	*2,240*	790	*287*	*2,699*	1,282	*695*	*2,056*	256	*69*	*617*
Nicaragua	16	*10*	*24*	8,110	*5,747*	*11,461*	1,890	*1,253*	*2,539*	803	*284*	*2,837*	1,871	*1,241*	*2,513*	374	*124*	*754*
Niger	115	*74*	*176*	7,388	*5,225*	*10,655*	2,612	*1,775*	*3,345*	638	*226*	*2,302*	2,257	*1,534*	*2,890*	451	*153*	*867*
Nigeria	109	*69*	*172*	7,125	*5,367*	*9,593*	2,875	*1,633*	*4,407*	412	*155*	*1,386*	1,662	*944*	*2,547*	332	*94*	*764*
Pakistan	33	*14*	*77*	8,642	*6,483*	*10,916*	1,358	*517*	*3,084*	707	*265*	*2,232*	1,111	*423*	*2,522*	222	*42*	*757*
Papua New Guinea	30	*26*	*34*	8,399	*6,059*	*11,696*	1,601	*941*	*2,304*	711	*256*	*2,474*	1,354	*796*	*1,949*	271	*80*	*585*
Republic of Moldova	1	*1*	*1*	8,453	*5,995*	*11,829*	1,547	*1,005*	*2,171*	805	*285*	*2,815*	1,473	*957*	*2,067*	295	*96*	*620*
Rwanda	44	*21*	*90*	7,052	*5,415*	*9,225*	2,948	*1,585*	*4,775*	695	*267*	*2,274*	2,907	*1,562*	*4,708*	581	*156*	*1,412*
Sao Tome and Principe	20	*12*	*32*	7,552	*5,226*	*11,060*	2,448	*1,774*	*2,940*	742	*257*	*2,715*	2,404	*1,742*	*2,887*	481	*174*	*866*
Senegal	39	*29*	*53*	7,575	*5,274*	*11,035*	2,425	*1,726*	*2,965*	726	*253*	*2,643*	2,323	*1,654*	*2,840*	465	*165*	*852*
Sierra Leone	101	*80*	*129*	7,365	*5,222*	*10,600*	2,635	*1,778*	*3,400*	701	*249*	*2,523*	2,508	*1,692*	*3,237*	502	*169*	*971*
Solomon Islands	11	*7*	*20*	8,343	*5,988*	*11,706*	1,657	*1,012*	*2,294*	816	*293*	*2,862*	1,620	*990*	*2,244*	324	*99*	*673*
Somalia	114	*77*	*168*	7,969	*5,682*	*11,292*	2,031	*1,318*	*2,708*	419	*149*	*1,485*	1,068	*693*	*1,425*	214	*69*	*427*
South Sudan	82	*42*	*162*	7,487	*5,228*	*10,898*	2,513	*1,772*	*3,102*	479	*167*	*1,744*	1,609	*1,134*	*1,986*	322	*113*	*596*
Sri Lanka	1	*0*	*2*	8,491	*6,018*	*11,983*	1,509	*982*	*2,017*	849	*301*	*2,996*	1,509	*982*	*2,017*	302	*98*	*605*
Sudan	60	*45*	*81*	8,185	*5,924*	*11,351*	1,815	*1,076*	*2,649*	799	*289*	*2,770*	1,771	*1,050*	*2,585*	354	*105*	*776*
Tajikistan	31	*20*	*47*	8,544	*6,053*	*11,959*	1,456	*947*	*2,041*	829	*294*	*2,900*	1,412	*919*	*1,980*	282	*92*	*594*
Timor-Leste	35	*17*	*69*	7,972	*5,862*	*10,855*	2,028	*1,138*	*3,145*	639	*235*	*2,176*	1,627	*912*	*2,522*	325	*91*	*757*
Togo	66	*42*	*104*	7,117	*5,377*	*9,541*	2,883	*1,623*	*4,459*	669	*253*	*2,242*	2,710	*1,526*	*4,191*	542	*153*	*1,257*
Uganda	32	*31*	*34*	7,442	*5,268*	*10,711*	2,558	*1,732*	*3,289*	662	*234*	*2,383*	2,277	*1,542*	*2,927*	455	*154*	*878*
Ukraine	1	*0*	*2*	8,412	*5,860*	*11,983*	1,588	*1,140*	*2,017*	841	*293*	*2,996*	1,588	*1,140*	*2,017*	318	*114*	*605*
United Republic of Tanzania	29	*20*	*44*	7,648	*5,462*	*10,870*	2,352	*1,538*	*3,130*	751	*268*	*2,669*	2,310	*1,511*	*3,074*	462	*151*	*922*
Uzbekistan	7	*1*	*40*	8,678	*6,151*	*12,123*	1,322	*849*	*1,877*	859	*304*	*3,000*	1,308	*840*	*1,858*	262	*84*	*558*
Viet Nam	5	*1*	*31*	7,832	*5,564*	*11,231*	2,168	*1,436*	*2,769*	732	*260*	*2,622*	2,025	*1,341*	*2,586*	405	*134*	*776*
Yemen	43	*21*	*90*	7,906	*5,699*	*11,085*	2,094	*1,301*	*2,915*	639	*230*	*2,239*	1,692	*1,051*	*2,355*	338	*105*	*706*
Zambia	51	*46*	*58*	7,445	*5,227*	*10,795*	2,555	*1,773*	*3,205*	666	*234*	*2,413*	2,284	*1,585*	*2,865*	457	*159*	*859*
Zimbabwe	54	*46*	*63*	7,433	*5,228*	*10,763*	2,567	*1,772*	*3,237*	727	*256*	*2,632*	2,511	*1,733*	*3,166*	502	*173*	*950*

* Mortality rates reflect year 2015 without rotavirus vaccination. These rates are assumed to decline overtime in the absence of vaccination (consistent with overall under-5 mortality declines).

2. Additional data inputs and sources used to model vaccine program costs

	Base case	Low	High	Source
International handling (% of vaccine price)	3·5%	1·4%	5%	[46]
International transportation (% of vaccine price)	6%	2%	15%	Assumption
Incremental delivery cost per dose for low-income countries	$1·25	$1	$2·50	[45]
Incremental delivery cost per dose for lower- and upper-middle income countries	$1·86	$1	$2·50	[45]
Vaccine wastage rate				
ROTARIX	5%	1%	10%	[41]
RotaTeq	5%	1%	10%	
ROTAVAC	25%	10%	50%	
ROTASIIL	10%	5%	15%	
Safe disposal bag (100 doses capacity)	$0·8	-	-	

3. Additional parameters to model burden of disease

	Base case	Low	High	Source
**Disability weights for DALY calculation**				
Rotavirus (non-severe) case	0·188	0·125	0·264	[36]
Rotavirus severe case	0·247	0·164	0·348	
**Mean duration of illness (in days)**				
Rotavirus (non-severe) case	4	3	7	[37]
Rotavirus severe case	6	3	7	

4. Modeled rotavirus treatment costs for 73 countries (in 2015 US$)

	Average cost per clinic visit				Average cost per hospital admission			
	Total	Direct Medical	Direct Non-Medical	Indirect	Total	Direct Medical	Direct Non-Medical	Indirect
**All 73 countries**								
Mean	16.81	6.71	9.02	1.08	90.68	71.59	14.75	4.34
Median	13.24	5.36	7.21	0.83	46.70	36.07	7.43	3.32
Min	9.56	4.00	5.38	0.19	16.64	13.17	2.71	0.76
Max	44.77	16.86	22.67	5.24	337.52	262.48	54.08	20.96
Afghanistan	10.06	4.12	5.54	0.41	19.21	14.58	3.00	1.63
Angola	29.48	11.37	15.30	2.81	224.48	176.81	36.43	11.24
Armenia	25.61	9.90	13.32	2.39	185.62	145.98	30.08	9.56
Azerbaijan	30.40	11.36	15.28	3.77	268.94	210.51	43.37	15.06
Bangladesh	12.26	4.87	6.56	0.83	37.03	27.95	5.76	3.32
Benin	12.83	5.25	7.06	0.52	41.44	32.63	6.72	2.09
Bhutan	20.39	7.92	10.65	1.82	131.33	102.86	21.19	7.28
Bolivia (Plurinational State of)	27.63	10.88	14.64	2.11	246.17	197.12	40.62	8.43
Burkina Faso	12.02	4.96	6.66	0.40	34.81	27.52	5.67	1.62
Burundi	9.56	4.00	5.38	0.19	16.64	13.17	2.71	0.76
Cambodia	13.16	5.27	7.09	0.79	46.70	36.09	7.44	3.17
Cameroon	14.99	6.04	8.12	0.83	61.42	48.16	9.92	3.33
Central African Republic	11.28	4.72	6.34	0.22	27.40	21.98	4.53	0.89
Chad	13.08	5.35	7.20	0.53	42.71	33.65	6.93	2.13
Comoros	12.92	5.30	7.13	0.49	39.88	31.44	6.48	1.97
Congo	26.79	10.88	14.64	1.27	242.81	197.12	40.62	5.07
Côte d'Ivoire	26.48	10.88	14.64	0.96	241.57	197.12	40.62	3.83
Cuba	44.77	16.86	22.67	5.24	337.52	262.48	54.08	20.96

Dem· People's Republic of Korea	11.98	4.92	6.62	0.44	36.48	28.79	5.93	1.76
Democratic Republic of the Congo	11.86	4.92	6.62	0.31	35.97	28.79	5.93	1.25
Djibouti	16.91	6.64	8.93	1.33	76.52	59.03	12.16	5.33
Eritrea	9.82	4.03	5.42	0.37	17.67	13.42	2.76	1.49
Ethiopia	10.54	4.31	5.80	0.42	23.61	18.17	3.74	1.70
Gambia	11.87	4.92	6.62	0.32	36.01	28.79	5.93	1.29
Georgia	23.28	8.83	11.88	2.57	153.89	119.06	24.53	10.29
Ghana	13.75	5.46	7.35	0.94	47.89	36.59	7.54	3.75
Guinea	10.92	4.50	6.06	0.36	26.19	20.51	4.23	1.46
Guinea-Bissau	11.68	4.81	6.47	0.39	31.85	25.11	5.17	1.57
Guyana	27.91	10.70	14.39	2.83	172.52	133.67	27.54	11.31
Haiti	13.24	5.41	7.27	0.56	41.22	32.32	6.66	2.24
Honduras	20.67	8.07	10.86	1.73	121.16	94.72	19.52	6.93
India	15.46	6.13	8.24	1.09	72.46	56.46	11.63	4.37
Indonesia	19.48	7.33	9.86	2.29	109.88	83.51	17.21	9.17
Kenya	13.16	5.21	7.00	0.94	43.29	32.76	6.75	3.77
Kiribati	21.99	8.96	12.05	0.98	119.81	96.10	19.80	3.90
Kyrgyzstan	26.28	10.88	14.64	0.76	240.76	197.12	40.62	3.02
Lao People's Democratic Republic	26.77	10.88	14.64	1.25	242.72	197.12	40.62	4.98
Lesotho	14.46	5.85	7.87	0.73	54.73	42.95	8.85	2.92
Liberia	10.43	4.32	5.80	0.31	21.04	16.41	3.38	1.25
Madagascar	10.38	4.31	5.80	0.28	22.71	17.92	3.69	1.10
Malawi	11.39	4.75	6.39	0.25	28.49	22.78	4.69	1.02
Mali	11.99	4.90	6.59	0.50	33.98	26.53	5.47	1.98
Mauritania	14.19	5.65	7.60	0.94	54.89	42.40	8.74	3.76
Mongolia	19.18	7.02	9.44	2.72	102.88	76.29	15.72	10.87
Mozambique	11.29	4.66	6.26	0.36	27.84	21.88	4.51	1.45
Myanmar	12.78	5.11	6.87	0.80	38.19	29.03	5.98	3.18
Nepal	11.39	4.64	6.24	0.51	30.48	23.59	4.86	2.04
Nicaragua	17.61	6.90	9.28	1.43	91.89	71.45	14.72	5.72
Niger	10.38	4.32	5.81	0.25	21.31	16.85	3.47	0.98
Nigeria	16.59	6.29	8.47	1.83	71.66	53.35	10.99	7.32
Pakistan	13.02	5.13	6.91	0.98	47.07	35.77	7.37	3.93
Papua New Guinea	19.63	7.71	10.37	1.55	99.30	77.19	15.90	6.21
Republic of Moldova	26.79	10.88	14.64	1.27	242.80	197.12	40.62	5.06
Rwanda	11.91	4.87	6.56	0.48	33.21	25.95	5.35	1.91
Sao Tome and Principe	26.67	10.88	14.64	1.14	242.31	197.12	40.62	4.57
Senegal	14.21	5.80	7.80	0.62	53.78	42.55	8.77	2.46
Sierra Leone	11.13	4.56	6.13	0.45	27.88	21.64	4.46	1.79
Solomon Islands	21.61	8.65	11.64	1.33	120.87	95.82	19.74	5.30
Somalia	11.92	4.92	6.62	0.38	36.23	28.79	5.93	1.50
South Sudan	12.04	4.92	6.62	0.50	36.72	28.79	5.93	2.00
Sri Lanka	19.85	7.32	9.84	2.69	116.37	87.57	18.04	10.76
Sudan	16.52	6.34	8.52	1.65	73.10	55.13	11.36	6.62
Tajikistan	13.14	5.33	7.17	0.63	46.03	36.07	7.43	2.54
Timor-Leste	26.36	10.88	14.64	0.83	241.07	197.12	40.62	3.33
Togo	11.24	4.63	6.23	0.38	27.90	21.86	4.50	1.53
Uganda	11.73	4.80	6.45	0.48	32.46	25.31	5.22	1.93
Ukraine	23.22	9.28	12.49	1.45	173.89	139.37	28.72	5.79
United Republic of Tanzania	12.14	4.92	6.62	0.60	37.13	28.79	5.93	2.41

Uzbekistan	15.79	6.11	8.22	1.46	71.72	54.62	11.25	5.84
Viet Nam	26.97	10.88	14.64	1.45	243.52	197.12	40.62	5.78
Yemen	12.51	4.92	6.62	0.96	38.57	28.79	5.93	3.85
Zambia	13.46	5.36	7.21	0.89	44.99	34.34	7.07	3.58
Zimbabwe	12.18	4.92	6.62	0.63	37.25	28.79	5.93	2.53

5. Probabilistic Analysis

Parameter	Base case (95% CI)	Probability distribution	Source
**Population projections for the 2015 birth cohort**			
Population by single age/year between birth and 5·0 years	Country-specific	Beta-PERT (mid = UNPOP medium variant, range = UNPOP low/high variant)	[1]
**Disease burden estimates**			
RVGE incidence rate <5 years<br>Non-severe RVGE visits<br>Severe RVGE visits<br>Severe RVGE hospitalizations	Regional-specific	Beta-PERT (mid = regional value, range = reported uncertainty range for RVGE incidence, proportion range for treatment seeking rate:<br>10%-30% for hospitalizations, 5%-25% for clinic visits)	[2 -8]
Severe RVGE mortality rate <5 years	Country-specific	Beta-PERT (mid = Log transformed mean of 3 sources of country estimates, range = 95% CI)	[9-11]
**Age distribution of RVGE deaths**			
Log Logistic scale parameter	Country-specific	Beta-PERT (mid = best fit for country/U5MR stratum, range = 95% CI for country/U5MR stratum)	[12]
**Disability weights for calcualting DALYs**			
Percentage of healthy time lost whilst living with the disease			
Non Severe RVGE	18·8% (12·5-26·4)	Beta-PERT (mid = Salomon, GBD 2013, moderate diarhea, range = Salomon, GBD 2013, moderate diarrhea, 95% uncetainty interval)	[13]
Severe RVGE	24·7% (16·4-34·8)	Beta-PERT (mid = Salomon, GBD 2013, severe diarhea, range = Salomon, GBD 2013, severe diarhea, 95% uncetainty interval)	
Average duration of illness			
Non Severe RVGE	4 days (3-7)	Beta-PERT (mid = CDC, range = CDC)	[14]
Severe RVGE	6 days (3-7))	Beta-PERT (mid = CDC, range = CDC)	
**Vaccine coverage**			
Doses with DTP1, DTP2, DTP3	Country-specific	Beta-PERT (mid = WUENIC 2015, range = WUENIC 2015 +/-10%)	[15]
**Vaccine timeliness**			
Log Logistic scale parameter	Country-specific	Beta-PERT (mid = best fit for country or schedule stratum, range = median age +/-10%)	[16]
**Initial efficacy against RVGE mortality (2wks after dose administered)**			
Low mortality	99·6% (99·4-99·7)	Beta (alpha = 4918·07, beta = 19·83, [A] = 0%, [B] = 100%)	
Medium mortality	91·4% (89·8-92·7)	Beta (alpha = 1394·26, beta = 131·74, [A] = 0%, [B] = 100%)	[17]
High mortality	78·9% (75·5-82·3)	Beta (alpha = 723·19, beta = 98·31, [A] = 0%, [B] = 100%)	
**Mean duration of vaccine efficacy in months**			
Low mortality	176·8 (114·7-268·0)	Gamma (alpha = 21·82, beta = 8·41)	
Medium mortality	121·9 (81·3-182·4)	Gamma (alpha = 24·01, beta = 5·28)	[17]
High mortality	13·2 (9·2-20·5)	Gamma (alpha = 50·75, beta = 0·17)	
**Relative efficacy of 1 dose versus 2/3 doses**			
Low- and middle-income countries (LMICs)	0·63 (0·51-0·79)	Beta (alpha = 27·05, beta = 15·68, [A] = 0, [B] = 1)	[17]
**Incremental health system costs**			
Low income countries	$1·25 (1-2·50)	Beta-PERT (mid = ICAN data, range based on authors assumptions)	
Middle income countries	$1·86 (1-2·50)	Beta-PERT (mid = ICAN data, range based on authors assumptions)	[18]
**International handling**	3·5% (1·4-5)	Beta-PERT (mid = UNICEF, range based on min and max from UNICEF)	
**International delivery**	6% (2-15)	Beta-PERT (mid = literature data, range based on assumptions)	
**Vaccine wastage**			
ROTARIX, RotaTeq	5% (1-10)	Beta-PERT	
ROTAVAC	25% (10-50)	Beta-PERT (mid = India MoH, low range based on assumtpion, high range based on Gavi recommedation)	[19]
ROTASIIL	10% (5-15)		
**Treatment costs**			

Cost per clinic visit	Country-specific (+/- 50%)	Beta-PERT (mid = modeled data from WHO CHOICE, range = modeled data from	
Cost per hospitalization	Country-specific (+/- 50%)	WHO CHOICE +/- 50%)	[20]

1 United Nations DESA/Population Division. World Population Prospects 2017. 2017. https://population.un.org/wpp/ (accessed Feb 7, 2018).

2 Yen C, Armero Guardado JA, Alberto P, et al. Decline in rotavirus hospitalizations and health care visits for childhood diarrhea following rotavirus vaccination in El Salvador. Pediatr Infect Dis J 2011; 30(1 Suppl): S6-S10.

3 Bahl R, Ray P, Subodh S, et al. Incidence of severe rotavirus diarrhea in New Delhi, India, and G and P types of the infecting rotavirus strains. J Infect Dis 2005; 192 Suppl 1: S114-9.

4. Omore R, Khagayi S, Ogwel B, et al. Rates of hospitalization and death for all-cause and rotavirus acute gastroenteritis before rotavirus vaccine introduction in Kenya, 2010-2013. BMC Infect Dis 2019; 19(1): 47.

5. Rheingans RD, Constenla D, Antil L, Innis BL, Breuer T. Economic and health burden of rotavirus gastroenteritis for the 2003 birth cohort in eight Latin American and Caribbean countries. Rev Panam Salud Publica 2007; 21(4): 192-204.

6. Carlos CC, Inobaya MT, Bresee JS, et al. The burden of hospitalizations and clinic visits for rotavirus disease in children aged <5 years in the Philippines. J Infect Dis 2009; 200 Suppl 1: S174-81.

7. Fischer TK, Anh DD, Antil L, et al. Health care costs of diarrheal disease and estimates of the cost-effectiveness of rotavirus vaccination in Vietnam. J Infect Dis 2005; 192(10): 1720-6.

8. Parashar UD, Hummelman EG, Bresee JS, Miller MA, Glass RI. Global illness and deaths caused by rotavirus disease in children. *Emerg Infect Dis* 2003; 9: 565–72.

9 GBD 2013 Mortality and Causes of Death Collaborators. Global, regional, and national age-sex specific all-cause and cause-specific mortality for 240 causes of death, 1990-2013: a systematic analysis for the Global Burden of Disease Study 2013. *Lancet* 2015; 385: 117–71.

10 Vos T, Barber R, Phillips DE, Lopez AD, Murray CJL. Causes of child death: comparison of MCEE and GBD 2013 estimates – Authors' reply. *Lancet* 2015; 385: 2462–4.

11 Tate JE, Burton AH, Boschi-Pinto C, Parashar UD. Global, Regional, and National Estimates of Rotavirus Mortality in Children <5 Years of Age, 2000–2013. *Clin Infect Dis* 2016; 62: S96–105.

12 Hasso-Agopsowicz M, Ladva CN, Lopman B, et al. Global review of the age distribution of rotavirus disease in children aged <5 years before the introduction of rotavirus vaccination. *Clin Infect Dis* 2019; published online Jan 28. DOI:10.1093/cid/ciz060. 36 Salomon JA, Haagsma JA, Davis A, et al. Disability weights for the Global Burden of Disease 2013 study. *Lancet Glob Heal* 2015; 3: e712–23.

13 Salomon JA, Haagsma JA, Davis A, et al. Disability weights for the Global Burden of Disease 2013 study. *Lancet Glob Heal* 2015; 3: e712–23.

14 Centers for Disease Control and Prevention. Rotavirus. In: Hamborsky J, Kroger A, Wolfe C, eds. Epidemiology and Prevention of Vaccine Preventable Diseases, 13th ed. Washington D.C.: Public Health Foundation, 2015. https://www.cdc.gov/vaccines/pubs/pinkbook/rota.html (accessed May 21, 2019).

15 World Health Organization. WHO/UNICEF estimates of national immunization coverage. 2017. https://www.who.int/immunization/monitoring_surveillance/routine/coverage/en/index4.html (accessed Feb 7, 2018).

16 Clark A, Tate J, Parashar U, et al. Mortality reduction benefits and intussusception risks of rotavirus vaccination in 135 low-income and middle-income countries: a modelling analysis of current and alternative schedules. Lancet Glob Health 7: e1541–52.

17 Clark A, van Zandvoort K, Flasche S, at al. Efficacy of live oral rotavirus vaccines by duration of follow-up: a meta-regression of randomised controlled trials. Lancet Infect Dis 2019;19: 717–27. http://dx.doi.org/10.1016/S1473-3099(19)30126-4

18 Immunization Costing Action Network (ICAN). Unit Cost Repository for Immunization Delivery. 2018. https://immunizationeconomics.org/ican-idcc (accessed April 13, 2018).

19 Gavi The Vaccine Alliance. Gavi, the Vaccine Alliance Co-financing Policy Version 2.0. 2016 https://www.gavi.org/library/gavi-documents/policies/gavi-co-financing-policy/ (accessed May 21, 2019).

20 Baral R, Nonvignon J, Debellut F, Agyemang SA, Clark A, Pecenka C. Cost of illness for childhood diarrhoea in low- and middle-income countries: a systematic review of evidence and modelled estimates. (Unpublished).

6. Vaccine price and co-financing inputs for all countries - alternative scenarios

Country	Vaccine preference	Vaccine price per dose US$	Average Co-financing per dose over the period 2018-2027 US$
Afghanistan	ROTARIX®	2·29	0·20
	ROTAVAC®	0·85	0·13
	ROTASIIL®	0·95	0·13
Angola	ROTARIX®	2·29	2·29
	ROTAVAC®	0·85	0·85
	ROTASIIL®	0·95	0·95
Armenia	ROTARIX®	2·29	2·29
	ROTAVAC®	0·85	0·85
	ROTASIIL®	0·95	0·95
Azerbaijan	ROTARIX®	6·20	6·20
	ROTAVAC®	0·85	0·85
	ROTASIIL®	0·95	0·95
Bangladesh	ROTARIX®	2·29	0·88
	ROTAVAC®	0·85	0·33
	ROTASIIL®	0·95	0·36
Benin	ROTARIX®	2·29	0·26
	ROTAVAC®	0·85	0·17
	ROTASIIL®	0·95	0·17
Bhutan	ROTARIX®	6·20	6·20
	ROTAVAC®	0·85	0·85
	ROTASIIL®	0·95	0·95
Bolivia	ROTARIX®	6·50	6·50
	ROTAVAC®	0·85	0·85
	ROTASIIL®	0·95	0·95
Burkina Faso	ROTARIX®	2·29	0·20
	ROTAVAC®	0·85	0·13
	ROTASIIL®	0·95	0·13
Burundi	ROTARIX®	2·29	0·20
	ROTAVAC®	0·85	0·13
	ROTASIIL®	0·95	0·13
Cambodia	ROTARIX®	2·29	0·47
	ROTAVAC®	0·85	0·18
	ROTASIIL®	0·95	0·20
Cameroon	ROTARIX®	2·29	0·90
	ROTAVAC®	0·85	0·33
	ROTASIIL®	0·95	0·37
CAR	ROTARIX®	2·29	0·20
	ROTAVAC®	0·85	0·13
	ROTASIIL®	0·95	0·13
Chad	ROTARIX®	2·29	0·20
	ROTAVAC®	0·85	0·13
	ROTASIIL®	0·95	0·13
Comoros	ROTARIX®	2·29	0·20

Country	Vaccine preference	Vaccine price per dose US$	Average Co-financing per dose over the period 2018-2027 US$
Lao PDR	ROTARIX®	2·29	1·88
	ROTAVAC®	0·85	0·70
	ROTASIIL®	0·95	0·78
Lesotho	ROTARIX®	2·29	0·46
	ROTAVAC®	0·85	0·17
	ROTASIIL®	0·95	0·19
Liberia	ROTARIX®	2·29	0·20
	ROTAVAC®	0·85	0·13
	ROTASIIL®	0·95	0·13
Madagascar	ROTARIX®	2·29	0·20
	ROTAVAC®	0·85	0·13
	ROTASIIL®	0·95	0·13
Malawi	ROTARIX®	2·29	0·20
	ROTAVAC®	0·85	0·13
	ROTASIIL®	0·95	0·13
Mali	ROTARIX®	2·29	0·20
	ROTAVAC®	0·85	0·13
	ROTASIIL®	0·95	0·13
Mauritania	ROTARIX®	2·29	0·55
	ROTAVAC®	0·85	0·20
	ROTASIIL®	0·95	0·23
Mongolia	ROTARIX®	6·20	6·20
	ROTAVAC®	0·85	0·85
	ROTASIIL®	0·95	0·95
Mozambique	ROTARIX®	2·29	0·20
	ROTAVAC®	0·85	0·13
	ROTASIIL®	0·95	0·13
Myanmar	ROTARIX®	2·29	0·71
	ROTAVAC®	0·85	0·26
	ROTASIIL®	0·95	0·29
Nepal	ROTARIX®	2·29	0·20
	ROTAVAC®	0·85	0·13
	ROTASIIL®	0·95	0·13
Nicaragua	ROTARIX®	6·50	5·98
	ROTAVAC®	0·85	0·78
	ROTASIIL®	0·95	0·87
Niger	ROTARIX®	2·29	0·20
	ROTAVAC®	0·85	0·13
	ROTASIIL®	0·95	0·13
Nigeria	ROTARIX®	2·29	1·39
	ROTAVAC®	0·85	0·51
	ROTASIIL®	0·95	0·58
Pakistan	ROTARIX®	2·29	1·25

	ROTAVAC®	0·85	0·13
	ROTASIIL®	0·95	0·13
Congo	ROTARIX®	2·29	2·29
	ROTAVAC®	0·85	0·85
	ROTASIIL®	0·95	0·95
Côte d'Ivoire	ROTARIX®	2·29	1·11
	ROTAVAC®	0·85	0·41
	ROTASIIL®	0·95	0·46
Cuba	ROTARIX®	6·50	6·50
	ROTAVAC®	0·85	0·85
	ROTASIIL®	0·95	0·95
Djibouti	ROTARIX®	2·29	0·53
	ROTAVAC®	0·85	0·20
	ROTASIIL®	0·95	0·22
DPR Korea	ROTARIX®	2·29	0·20
	ROTAVAC®	0·85	0·13
	ROTASIIL®	0·95	0·13
DR Congo	ROTARIX®	2·29	0·20
	ROTAVAC®	0·85	0·13
	ROTASIIL®	0·95	0·13
Eritrea	ROTARIX®	2·29	0·23
	ROTAVAC®	0·85	0·16
	ROTASIIL®	0·95	0·16
Ethiopia	ROTARIX®	2·29	0·21
	ROTAVAC®	0·85	0·14
	ROTASIIL®	0·95	0·14
Gambia	ROTARIX®	2·29	0·20
	ROTAVAC®	0·85	0·13
	ROTASIIL®	0·95	0·13
Georgia	ROTARIX®	2·29	2·29
	ROTAVAC®	0·85	0·85
	ROTASIIL®	0·95	0·95
Ghana	ROTARIX®	2·29	0·67
	ROTAVAC®	0·85	0·25
	ROTASIIL®	0·95	0·28
Guinea	ROTARIX®	2·29	0·20
	ROTAVAC®	0·85	0·13
	ROTASIIL®	0·95	0·13
Guinea-Bissau	ROTARIX®	2·29	0·20
	ROTAVAC®	0·85	0·13
	ROTASIIL®	0·95	0·13
Guyana	ROTARIX®	6·50	6·50
	ROTAVAC®	0·85	0·85
	ROTASIIL®	0·95	0·95
Haiti	ROTARIX®	6·50	0·20
	ROTAVAC®	0·85	0·13
	ROTASIIL®	0·95	0·13
Honduras	ROTARIX®	6·50	6·50
	ROTAVAC®	0·85	0·85
	ROTASIIL®	0·95	0·95
	ROTAVAC®	0·85	0·46
	ROTASIIL®	0·95	0·52
Papua New Guinea	ROTARIX®	2·29	2·10
	ROTAVAC®	0·85	0·78
	ROTASIIL®	0·95	0·87
Republic of Moldova	ROTARIX®	2·29	2·29
	ROTAVAC®	0·85	0·85
	ROTASIIL®	0·95	0·95
Rwanda	ROTARIX®	2·29	0·20
	ROTAVAC®	0·85	0·14
	ROTASIIL®	0·95	0·14
Sao Tome & P·	ROTARIX®	2·29	1·69
	ROTAVAC®	0·85	0·63
	ROTASIIL®	0·95	0·70
Senegal	ROTARIX®	2·29	0·20
	ROTAVAC®	0·85	0·13
	ROTASIIL®	0·95	0·13
Sierra Leone	ROTARIX®	2·29	0·20
	ROTAVAC®	0·85	0·13
	ROTASIIL®	0·95	0·13
Solomon Islands	ROTARIX®	2·29	1·93
	ROTAVAC®	0·85	0·72
	ROTASIIL®	0·95	0·80
Somalia	ROTARIX®	2·29	0·20
	ROTAVAC®	0·85	0·13
	ROTASIIL®	0·95	0·13
South Sudan	ROTARIX®	2·29	0·20
	ROTAVAC®	0·85	0·13
	ROTASIIL®	0·95	0·13
Sri Lanka	ROTARIX®	6·20	6·20
	ROTAVAC®	0·85	0·85
	ROTASIIL®	0·95	0·95
Sudan	ROTARIX®	2·29	1·34
	ROTAVAC®	0·85	0·50
	ROTASIIL®	0·95	0·55
Tajikistan	ROTARIX®	2·29	0·60
	ROTAVAC®	0·85	0·22
	ROTASIIL®	0·95	0·25
Timor-Leste	ROTARIX®	6·20	6·20
	ROTAVAC®	0·85	0·85
	ROTASIIL®	0·95	0·95
Togo	ROTARIX®	2·29	0·20
	ROTAVAC®	0·85	0·13
	ROTASIIL®	0·95	0·13
Uganda	ROTARIX®	2·29	0·20
	ROTAVAC®	0·85	0·13
	ROTASIIL®	0·95	0·13
Ukraine	ROTARIX®	6·20	6·20
	ROTAVAC®	0·85	0·85
	ROTASIIL®	0·95	0·95

India	ROTARIX®	2·29	2·29
	ROTAVAC®	0·85	0·85
	ROTASIIL®	0·95	0·95
Indonesia	ROTARIX®	6·20	6·20
	ROTAVAC®	0·85	0·85
	ROTASIIL®	0·95	0·95
Kenya	ROTARIX®	2·29	0·87
	ROTAVAC®	0·85	0·32
	ROTASIIL®	0·95	0·36
Kiribati	ROTARIX®	6·20	6·20
	ROTAVAC®	0·85	0·85
	ROTASIIL®	0·95	0·95
Kyrgyzstan	ROTARIX®	2·29	0·65
	ROTAVAC®	0·85	0·24
	ROTASIIL®	0·95	0·27
United Republic of Tanzania	ROTARIX®	2·29	0·36
	ROTAVAC®	0·85	0·24
	ROTASIIL®	0·95	0·24
Uzbekistan	ROTARIX®	2·29	2·29
	ROTAVAC®	0·85	0·85
	ROTASIIL®	0·95	0·95
Viet Nam	ROTARIX®	2·29	2·29
	ROTAVAC®	0·85	0·85
	ROTASIIL®	0·95	0·95
Yemen	ROTARIX®	2·29	0·68
	ROTAVAC®	0·85	0·25
	ROTASIIL®	0·95	0·28
Zambia	ROTARIX®	2·29	0·74
	ROTAVAC®	0·85	0·28
	ROTASIIL®	0·95	0·31
Zimbabwe	ROTARIX®	2·29	0·20
	ROTAVAC®	0·85	0·13
	ROTASIIL®	0·95	0·13

7. Results by country for alternative scenarios (all countries using rotavirus vaccine from 2018 to 2027)

Countries	Vaccine	Number of Fully Immunized Child	Averted RV cases	Averted RV deaths	Total healthcare costs averted government perspective*	Vaccine program costs*	Vaccine program costs with Gavi subsidy*	Cost per DALY averted govt. perspective*
Afghanistan	ROTARIX®	7,762,176	1,580,540	7,305	$1,790,959	$54,638,902	$20,878,029	$102
	ROTAVAC®	6,356,393	1,773,862	8,198	$2,009,196	$50,995,752	$29,655,963	$132
	ROTASIIL®	6,356,393	1,773,862	8,198	$2,009,196	$49,255,490	$28,990,961	$128
Angola	ROTARIX®	9,358,710	2,242,237	30,528	$21,333,511	$77,669,464	$77,669,464	$74
	ROTAVAC®	8,296,959	2,481,298	33,783	$23,604,682	$76,353,426	$76,353,426	$62
	ROTASIIL®	8,296,959	2,481,298	33,783	$23,604,682	$74,262,492	$74,262,492	$60
Armenia	ROTARIX®	307,794	106,670	9	$473,387	$2,499,384	$2,499,384	$4,337
	ROTAVAC®	283,678	110,379	9	$489,905	$2,564,715	$2,564,715	$4,292
	ROTASIIL®	283,678	110,379	9	$489,905	$2,494,434	$2,494,434	$4,146
Azerbaijan	ROTARIX®	1,331,157	230,840	165	$1,249,383	$21,223,960	$21,223,960	$4,233
	ROTAVAC®	1,296,082	256,505	183	$1,388,389	$10,954,903	$10,954,903	$1,824
	ROTASIIL®	1,296,082	256,505	183	$1,388,389	$10,654,872	$10,654,872	$1,767
Bangladesh	ROTARIX®	28,123,647	5,833,670	5,519	$11,432,203	$224,570,680	$139,018,609	$813
	ROTAVAC®	27,821,356	6,381,993	6,038	$12,508,907	$231,188,876	$171,162,261	$924
	ROTASIIL®	27,821,356	6,381,993	6,038	$12,508,907	$224,858,236	$168,951,095	$911
Benin	ROTARIX®	3,519,063	830,088	5,999	$2,192,702	$24,316,608	$9,672,091	$50
	ROTAVAC®	3,409,188	915,805	6,618	$2,419,236	$23,113,488	$13,917,319	$69
	ROTASIIL®	3,409,188	915,805	6,618	$2,419,236	$22,326,095	$13,537,869	$67
Bhutan	ROTARIX®	133,463	28,184	15	$118,366	$2,134,759	$2,134,759	$4,559
	ROTAVAC®	130,982	31,051	16	$130,410	$1,101,703	$1,101,703	$1,993
	ROTASIIL®	130,982	31,051	16	$130,410	$1,071,531	$1,071,531	$1,931
Bolivia (Plurinational State of)	ROTARIX®	2,458,502	552,970	820	$4,601,265	$40,490,928	$40,490,928	$1,599
	ROTAVAC®	2,434,970	618,446	917	$5,145,490	$20,176,743	$20,176,743	$599

	ROTASIIL®	2,434,970	618,446	917	$5,145,490	$19,624,199	$19,624,199	$577
Burkina Faso	ROTARIX®	7,100,998	1,595,662	10,176	$4,572,707	$48,962,773	$18,709,128	$55
	ROTAVAC®	6,938,849	1,741,437	11,105	$4,990,861	$46,597,089	$27,096,388	$79
	ROTASIIL®	6,938,849	1,741,437	11,105	$4,990,861	$45,009,804	$26,491,697	$77
Burundi	ROTARIX®	4,369,163	975,496	6,008	$1,609,641	$30,075,387	$11,492,083	$66
	ROTAVAC®	4,299,965	1,065,399	6,562	$1,758,198	$28,705,014	$16,692,071	$91
	ROTASIIL®	4,299,965	1,065,399	6,562	$1,758,198	$27,727,215	$16,319,576	$89
Cambodia	ROTARIX®	3,130,641	636,681	593	$1,615,454	$24,997,301	$13,204,131	$694
	ROTAVAC®	3,093,143	693,787	646	$1,760,655	$25,723,851	$17,452,512	$863
	ROTASIIL®	3,093,143	693,787	646	$1,760,655	$25,019,452	$17,315,754	$855
Cameroon	ROTARIX®	7,659,052	1,813,529	13,867	$7,230,672	$61,673,034	$39,524,515	$93
	ROTAVAC®	7,329,381	1,975,639	15,107	$7,877,224	$62,574,926	$47,259,788	$104
	ROTASIIL®	7,329,381	1,975,639	15,107	$7,877,224	$60,861,379	$46,597,280	$102
Central African Republic	ROTARIX®	945,985	247,845	3,149	$388,046	$7,106,568	$2,715,485	$30
	ROTAVAC®	733,714	269,033	3,419	$421,169	$6,240,284	$3,628,809	$38
	ROTASIIL®	733,714	269,033	3,419	$421,169	$6,027,607	$3,547,718	$37
Chad	ROTARIX®	3,296,107	703,117	11,176	$1,260,821	$24,671,508	$9,427,211	$30
	ROTAVAC®	2,812,216	766,128	12,178	$1,373,576	$22,253,447	$12,941,416	$40
	ROTASIIL®	2,812,216	766,128	12,178	$1,373,576	$21,493,685	$12,650,864	$39
Comoros	ROTARIX®	240,962	54,358	176	$139,814	$1,673,552	$639,480	$111
	ROTAVAC®	228,838	59,906	194	$154,074	$1,588,154	$923,524	$155
	ROTASIIL®	228,838	59,906	194	$154,074	$1,534,044	$902,903	$150
Congo	ROTARIX®	1,536,206	348,359	1,090	$4,065,847	$12,290,418	$12,290,418	$292
	ROTAVAC®	1,487,337	380,510	1,191	$4,441,457	$12,527,243	$12,527,243	$262
	ROTASIIL®	1,487,337	380,510	1,191	$4,441,457	$12,184,205	$12,184,205	$251
Côte d'Ivoire	ROTARIX®	8,300,070	1,986,436	9,045	$20,328,412	$68,545,450	$47,462,102	$123
	ROTAVAC®	7,677,006	2,171,684	9,889	$22,224,658	$68,486,281	$54,136,596	$133
	ROTASIIL®	7,677,006	2,171,684	9,889	$22,224,658	$66,610,458	$53,245,556	$129
Cuba	ROTARIX®	1,127,021	502,734	18	$6,119,296	$18,565,074	$18,565,074	$7,544
	ROTAVAC®	1,116,234	508,721	19	$6,191,925	$9,251,615	$9,251,615	$1,833
	ROTASIIL®	1,116,234	508,721	19	$6,191,925	$8,998,257	$8,998,257	$1,681
Dem. People's Republic of Korea	ROTARIX®	3,334,760	1,180,956	758	$2,151,598	$23,000,045	$8,788,530	$300
	ROTAVAC®	3,272,406	1,214,173	779	$2,212,329	$22,063,877	$12,830,293	$467
	ROTASIIL®	3,272,406	1,214,173	779	$2,212,329	$21,312,178	$12,543,852	$455
Dem. Rep. of the Congo	ROTARIX®	27,853,068	6,592,422	48,082	$16,933,114	$191,321,705	$73,105,790	$46
	ROTAVAC®	27,297,657	7,268,535	53,013	$18,669,915	$183,426,127	$106,663,660	$66
	ROTASIIL®	27,297,657	7,268,535	53,013	$18,669,915	$177,176,708	$104,282,116	$64
Djibouti	ROTARIX®	178,289	40,238	121	$127,781	$1,441,579	$786,340	$212
	ROTAVAC®	169,808	44,216	133	$140,413	$1,466,119	$1,012,010	$256
	ROTASIIL®	169,808	44,216	133	$140,413	$1,425,966	$1,003,021	$253
Eritrea	ROTARIX®	1,539,050	261,276	770	$421,978	$10,611,584	$4,155,848	$188
	ROTAVAC®	1,502,333	287,766	848	$464,824	$10,153,019	$6,031,664	$254
	ROTASIIL®	1,502,333	287,766	848	$464,824	$9,807,115	$5,878,586	$247
Ethiopia	ROTARIX®	26,078,179	5,162,506	16,375	$7,580,039	$187,304,858	$72,057,918	$152
	ROTAVAC®	24,772,249	5,563,870	17,648	$8,169,938	$175,334,731	$102,561,211	$206
	ROTASIIL®	24,772,249	5,563,870	17,648	$8,169,938	$169,357,278	$100,180,790	$201
Gambia	ROTARIX®	818,934	167,343	533	$330,064	$5,663,362	$2,164,023	$137
	ROTAVAC®	792,715	184,210	587	$363,354	$5,391,343	$3,135,099	$188
	ROTASIIL®	792,715	184,210	587	$363,354	$5,207,671	$3,065,113	$183
Georgia	ROTARIX®	421,489	192,937	12	$738,532	$3,395,328	$3,395,328	$3,581

	ROTAVAC®	401,587	194,890	12	$745,984	$3,466,965	$3,466,965	$3,631
	ROTASIIL®	401,587	194,890	12	$745,984	$3,372,013	$3,372,013	$3,504
Ghana	ROTARIX®	8,085,885	1,775,985	6,344	$6,160,965	$64,090,055	$37,213,746	$192
	ROTAVAC®	8,040,271	1,931,569	6,899	$6,701,526	$66,138,805	$47,235,298	$230
	ROTASIIL®	8,040,271	1,931,569	6,899	$6,701,526	$64,327,732	$46,721,525	$227
Guinea	ROTARIX®	2,819,849	637,498	2,654	$1,079,517	$19,901,116	$7,604,400	$96
	ROTAVAC®	2,600,878	695,933	2,897	$1,178,462	$18,533,035	$10,777,108	$130
	ROTASIIL®	2,600,878	695,933	2,897	$1,178,462	$17,901,586	$10,536,462	$126
Guinea-Bissau	ROTARIX®	584,936	127,634	848	$320,995	$4,118,150	$1,573,583	$59
	ROTAVAC®	545,182	140,466	933	$353,237	$3,874,784	$2,253,224	$81
	ROTASIIL®	545,182	140,466	933	$353,237	$3,742,752	$2,202,899	$79
Guyana	ROTARIX®	141,621	30,965	46	$223,693	$2,335,070	$2,335,070	$1,736
	ROTAVAC®	141,157	34,842	51	$251,688	$1,163,671	$1,163,671	$666
	ROTASIIL®	141,157	34,842	51	$251,688	$1,131,805	$1,131,805	$643
Haiti	ROTARIX®	1,642,083	380,229	1,557	$609,246	$27,623,504	$4,702,378	$103
	ROTAVAC®	1,271,839	416,461	1,705	$667,090	$11,013,492	$6,404,662	$132
	ROTASIIL®	1,271,839	416,461	1,705	$667,090	$10,637,832	$6,261,230	$128
Honduras	ROTARIX®	1,920,553	422,385	391	$2,083,282	$31,797,350	$31,797,350	$2,667
	ROTAVAC®	1,899,458	477,368	442	$2,354,359	$15,766,012	$15,766,012	$1,065
	ROTASIIL®	1,899,458	477,368	442	$2,354,359	$15,334,291	$15,334,291	$1,031
India	ROTARIX®	216,885,047	41,643,699	95,794	$106,415,765	$1,741,279,657	$1,741,279,657	$648
	ROTAVAC®	210,355,220	46,083,379	106,007	$117,772,708	$1,786,444,633	$1,786,444,633	$597
	ROTASIIL®	210,355,220	46,083,379	106,007	$117,772,708	$1,737,518,835	$1,737,518,835	$580
Indonesia	ROTARIX®	40,066,627	7,788,645	10,823	$37,927,779	$665,220,834	$665,220,834	$2,159
	ROTAVAC®	35,606,711	8,613,368	11,969	$41,943,398	$329,048,931	$329,048,931	$894
	ROTASIIL®	35,606,711	8,613,368	11,969	$41,943,398	$320,037,163	$320,037,163	$866
Kenya	ROTARIX®	14,630,923	3,225,501	8,877	$8,233,918	$117,831,196	$73,903,636	$283
	ROTAVAC®	14,076,582	3,499,623	9,631	$8,934,959	$119,659,458	$89,255,279	$319
	ROTASIIL®	14,076,582	3,499,623	9,631	$8,934,959	$116,382,842	$88,065,224	$315
Kiribati	ROTARIX®	25,503	5,365	14	$23,395	$406,410	$406,410	$1,080
	ROTAVAC®	25,002	5,910	15	$25,776	$209,627	$209,627	$471
	ROTASIIL®	25,002	5,910	15	$25,776	$203,886	$203,886	$456
Kyrgyzstan	ROTARIX®	1,244,976	450,885	225	$2,806,002	$9,886,628	$5,696,035	$429
	ROTAVAC®	1,219,828	466,414	232	$2,902,866	$10,203,078	$7,256,136	$625
	ROTASIIL®	1,219,828	466,414	232	$2,902,866	$9,923,656	$7,178,955	$614
Lao People's Democratic Republic	ROTARIX®	1,186,648	237,521	1,326	$2,217,023	$9,984,407	$8,828,923	$195
	ROTAVAC®	1,066,947	265,434	1,481	$2,476,970	$10,225,933	$9,431,407	$183
	ROTASIIL®	1,066,947	265,434	1,481	$2,476,970	$9,945,424	$9,205,425	$177
Lesotho	ROTARIX®	550,446	126,078	594	$403,802	$4,419,060	$2,325,045	$133
	ROTAVAC®	535,914	138,148	650	$442,508	$4,513,444	$3,055,749	$165
	ROTASIIL®	535,914	138,148	650	$442,508	$4,389,853	$3,032,196	$164
Liberia	ROTARIX®	1,481,187	352,291	1,278	$587,456	$10,701,360	$4,089,089	$106
	ROTAVAC®	1,286,451	386,308	1,402	$644,131	$9,744,094	$5,666,261	$139
	ROTASIIL®	1,286,451	386,308	1,402	$644,131	$9,412,113	$5,539,752	$136
Madagascar	ROTARIX®	7,244,209	1,622,511	6,370	$2,465,080	$50,490,355	$19,292,831	$102
	ROTAVAC®	6,858,150	1,769,058	6,945	$2,687,765	$47,531,205	$27,640,007	$139
	ROTASIIL®	6,858,150	1,769,058	6,945	$2,687,765	$45,911,327	$27,022,399	$136
Malawi	ROTARIX®	6,138,614	1,487,654	6,147	$3,486,309	$42,520,140	$16,247,338	$81
	ROTAVAC®	5,956,350	1,622,659	6,705	$3,802,896	$40,318,447	$23,445,330	$114
	ROTASIIL®	5,956,350	1,622,659	6,705	$3,802,896	$38,945,043	$22,922,123	$111

Mali	ROTARIX®	6,184,258	1,403,622	7,001	$2,329,722	$44,878,632	$17,148,540	$84
	ROTAVAC®	5,370,633	1,528,415	7,623	$2,536,699	$40,776,423	$23,712,106	$111
	ROTASIIL®	5,370,633	1,528,415	7,623	$2,536,699	$39,386,585	$23,182,098	$108
Mauritania	ROTARIX®	1,200,458	270,702	1,537	$870,930	$9,887,444	$5,457,313	$118
	ROTAVAC®	1,083,368	298,162	1,692	$959,283	$9,801,262	$6,808,444	$136
	ROTASIIL®	1,083,368	298,162	1,692	$959,283	$9,532,809	$6,745,380	$135
Mongolia	ROTARIX®	598,514	229,016	130	$953,462	$9,477,638	$9,477,638	$2,218
	ROTAVAC®	598,514	234,615	133	$976,855	$4,904,458	$4,904,458	$997
	ROTASIIL®	598,514	234,615	133	$976,855	$4,770,160	$4,770,160	$963
Mozambique	ROTARIX®	10,006,557	2,270,541	9,103	$4,751,912	$70,827,392	$27,063,800	$97
	ROTAVAC®	9,083,237	2,496,548	10,009	$5,224,219	$66,153,609	$38,469,083	$132
	ROTASIIL®	9,083,237	2,496,548	10,009	$5,224,219	$63,899,240	$37,609,671	$128
Myanmar	ROTARIX®	8,362,868	1,789,483	4,287	$3,928,553	$67,119,696	$39,096,901	$315
	ROTAVAC®	8,102,780	2,040,478	4,888	$4,479,595	$68,695,435	$49,153,966	$351
	ROTASIIL®	8,102,780	2,040,478	4,888	$4,479,595	$66,814,203	$48,613,815	$347
Nepal	ROTARIX®	5,006,028	1,030,583	753	$1,410,178	$34,899,087	$13,335,264	$547
	ROTAVAC®	4,864,133	1,116,376	816	$1,527,722	$33,107,970	$19,252,409	$751
	ROTASIIL®	4,864,133	1,116,376	816	$1,527,722	$31,980,187	$18,822,774	$733
Nicaragua	ROTARIX®	1,092,186	384,373	460	$1,507,347	$18,081,624	$16,747,246	$1,186
	ROTAVAC®	1,076,241	398,380	477	$1,562,399	$8,987,481	$8,657,343	$533
	ROTASIIL®	1,076,241	398,380	477	$1,562,399	$8,741,357	$8,433,875	$516
Niger	ROTARIX®	8,811,793	2,098,720	19,320	$3,657,328	$63,969,987	$24,443,523	$43
	ROTAVAC®	7,564,498	2,292,625	21,106	$3,995,130	$57,772,728	$33,595,212	$56
	ROTASIIL®	7,564,498	2,292,625	21,106	$3,995,130	$55,804,490	$32,845,221	$55
Nigeria	ROTARIX®	41,627,190	9,756,871	84,218	$26,096,216	$352,784,094	$270,241,552	$120
	ROTAVAC®	35,492,367	10,650,771	91,934	$28,485,738	$342,230,664	$287,705,916	$116
	ROTASIIL®	35,492,367	10,650,771	91,934	$28,485,738	$332,854,877	$282,072,023	$114
Pakistan	ROTARIX®	37,967,843	8,098,797	23,446	$12,209,306	$308,853,983	$222,737,186	$343
	ROTAVAC®	35,797,028	8,801,555	25,481	$13,268,537	$312,302,252	$252,993,069	$359
	ROTASIIL®	35,797,028	8,801,555	25,481	$13,268,537	$303,748,817	$248,509,872	$353
Papua New Guinea	ROTARIX®	1,797,367	399,995	1,038	$1,281,048	$14,920,903	$14,150,660	$479
	ROTAVAC®	1,607,485	441,152	1,145	$1,412,872	$14,741,493	$14,223,924	$432
	ROTASIIL®	1,607,485	441,152	1,145	$1,412,872	$14,337,773	$13,855,723	$420
Republic of Moldova	ROTARIX®	319,744	114,889	9	$827,754	$2,576,006	$2,576,006	$3,433
	ROTAVAC®	310,551	118,846	10	$856,349	$2,629,527	$2,629,527	$3,365
	ROTASIIL®	310,551	118,846	10	$856,349	$2,557,521	$2,557,521	$3,229
Rwanda	ROTARIX®	3,585,413	715,749	2,539	$2,031,404	$24,601,057	$9,419,779	$112
	ROTAVAC®	3,567,212	772,472	2,741	$2,192,822	$23,604,645	$13,750,810	$162
	ROTASIIL®	3,567,212	772,472	2,741	$2,192,822	$22,800,584	$13,440,397	$158
Sao Tome and Principe	ROTARIX®	66,365	13,923	25	$156,108	$525,494	$439,991	$428
	ROTAVAC®	66,017	15,146	27	$169,848	$542,339	$482,195	$433
	ROTASIIL®	66,017	15,146	27	$169,848	$527,488	$471,471	$418
Senegal	ROTARIX®	5,356,154	1,078,428	3,025	$3,505,164	$36,891,487	$14,096,578	$134
	ROTAVAC®	5,241,869	1,174,071	3,293	$3,816,223	$35,205,747	$20,472,319	$194
	ROTASIIL®	5,241,869	1,174,071	3,293	$3,816,223	$34,006,423	$20,015,378	$188
Sierra Leone	ROTARIX	2,237,531	494,152	4,068	$1,096,614	$15,995,382	$6,111,983	$51
	ROTAVAC	2,034,879	542,830	4,469	$1,204,550	$14,848,401	$8,634,480	$69
	ROTASIIL	2,034,879	542,830	4,469	$1,204,550	$14,342,464	$8,441,648	$67
Solomon Islands	ROTARIX®	170,828	36,041	32	$163,413	$1,354,672	$1,220,651	$1,186
	ROTAVAC®	168,506	39,695	35	$179,991	$1,401,486	$1,307,173	$1,148

	ROTASIIL®	168,506	39,695	35	$179,991	$1,363,103	$1,275,262	$1,116
Somalia	ROTARIX®	3,098,083	723,053	6,774	$846,259	$22,315,011	$8,526,772	$46
	ROTAVAC®	2,731,238	791,252	7,413	$926,056	$20,377,550	$11,849,677	$60
	ROTASIIL®	2,731,238	791,252	7,413	$926,056	$19,683,313	$11,585,139	$58
South Sudan	ROTARIX®	1,380,059	331,863	2,167	$564,120	$10,148,222	$3,877,729	$61
	ROTAVAC®	1,160,690	362,015	2,364	$615,357	$9,089,747	$5,285,746	$79
	ROTASIIL®	1,160,690	362,015	2,364	$615,357	$8,780,072	$5,167,745	$77
Sri Lanka	ROTARIX®	2,895,874	1,298,752	68	$4,874,860	$46,092,520	$46,092,520	$8,690
	ROTAVAC®	2,868,156	1,315,209	69	$4,936,522	$23,848,677	$23,848,677	$3,938
	ROTASIIL®	2,868,156	1,315,209	69	$4,936,522	$23,195,577	$23,195,577	$3,802
Sudan	ROTARIX®	12,845,274	2,833,307	14,567	$8,823,965	$103,124,331	$77,184,096	$184
	ROTAVAC®	12,263,663	3,120,170	16,041	$9,716,887	$105,559,187	$87,496,743	$190
	ROTASIIL®	12,263,663	3,120,170	16,041	$9,716,887	$102,667,745	$85,844,880	$186
Tajikistan	ROTARIX®	2,291,676	515,367	1,320	$995,629	$18,273,646	$10,308,538	$265
	ROTAVAC®	2,233,694	567,532	1,454	$1,096,436	$18,809,006	$13,222,459	$314
	ROTASIIL®	2,233,694	567,532	1,454	$1,096,436	$18,293,874	$13,090,717	$310
Timor-Leste	ROTARIX®	418,352	90,188	220	$695,130	$6,785,755	$6,785,755	$1,047
	ROTAVAC®	391,577	99,103	242	$763,884	$3,416,235	$3,416,235	$415
	ROTASIIL®	391,577	99,103	242	$763,884	$3,322,673	$3,322,673	$400
Togo	ROTARIX®	2,416,422	540,639	3,061	$1,293,933	$16,683,379	$6,374,873	$67
	ROTAVAC®	2,356,941	589,551	3,338	$1,411,080	$15,873,336	$9,230,409	$94
	ROTASIIL®	2,356,941	589,551	3,338	$1,411,080	$15,332,620	$9,024,415	$91
Uganda	ROTARIX®	15,696,079	3,497,987	9,314	$7,773,885	$110,814,276	$42,343,158	$146
	ROTAVAC®	14,018,297	3,903,689	10,394	$8,674,297	$103,141,041	$59,977,570	$194
	ROTASIIL®	14,018,297	3,903,689	10,394	$8,674,297	$99,626,456	$58,637,889	$189
Ukraine	ROTARIX®	1,136,251	685,509	35	$3,871,135	$24,329,758	$24,329,758	$8,456
	ROTAVAC®	630,398	692,799	35	$3,911,990	$10,580,689	$10,580,689	$2,728
	ROTASIIL®	630,398	692,799	35	$3,911,990	$10,290,365	$10,290,365	$2,609
United Republic of Tanzania	ROTARIX®	22,592,798	5,235,956	12,673	$12,913,994	$154,758,157	$66,128,551	$160
	ROTAVAC®	22,333,962	5,747,996	13,912	$14,178,194	$148,109,240	$94,941,109	$221
	ROTASIIL®	22,333,962	5,747,996	13,912	$14,178,194	$143,064,043	$91,549,924	$212
Uzbekistan	ROTARIX®	5,772,626	1,169,250	747	$2,809,315	$45,978,443	$45,978,443	$1,979
	ROTAVAC®	5,649,493	1,296,604	828	$3,115,241	$47,567,256	$47,567,256	$1,837
	ROTASIIL®	5,649,493	1,296,604	828	$3,115,241	$46,264,496	$46,264,496	$1,784
Viet Nam	ROTARIX®	14,299,616	4,434,155	1,924	$42,052,345	$141,769,165	$141,769,165	$1,658
	ROTAVAC®	14,251,963	4,522,533	1,963	$42,895,925	$161,291,552	$161,291,552	$1,930
	ROTASIIL®	14,251,963	4,522,533	1,963	$42,895,925	$157,162,880	$157,162,880	$1,862
Yemen	ROTARIX®	6,182,800	1,383,818	5,075	$2,560,785	$62,941,149	$42,007,560	$303
	ROTAVAC®	5,888,548	1,516,019	5,560	$2,805,409	$69,972,295	$55,462,773	$369
	ROTASIIL®	5,888,548	1,516,019	5,560	$2,805,409	$68,509,341	$54,995,570	$365
Zambia	ROTARIX®	6,573,203	1,560,484	6,263	$4,279,137	$66,609,961	$45,525,340	$259
	ROTAVAC®	6,189,033	1,702,154	6,831	$4,667,364	$73,713,622	$59,159,941	$313
	ROTASIIL®	6,189,033	1,702,154	6,831	$4,667,364	$72,204,031	$58,649,132	$311
Zimbabwe	ROTARIX®	4,669,296	1,044,688	4,766	$2,768,787	$41,811,906	$21,871,830	$159
	ROTAVAC®	4,564,281	1,139,997	5,201	$3,021,715	$44,866,828	$32,015,344	$222
	ROTASIIL®	4,564,281	1,139,997	5,201	$3,021,715	$43,819,959	$31,616,030	$218

** Discounted value*
